# The Imperative for Innovative Enteric Nervous System–Intestinal Organoid Co-Culture Models: Transforming GI Disease Modeling and Treatment

**DOI:** 10.3390/cells13100820

**Published:** 2024-05-10

**Authors:** Cristina Llorente

**Affiliations:** Department of Medicine, University of California San Diego, MC0063, 9500 Gilman Drive, La Jolla, CA 92093, USA; allorenteizquierdo@health.ucsd.edu

**Keywords:** enteric nervous system (ENS), three-dimensional (3D), pluripotent stem cells (PSCs), intestinal organoids, myenteric neurons, submucosal neurons

## Abstract

This review addresses the need for innovative co-culture systems integrating the enteric nervous system (ENS) with intestinal organoids. The breakthroughs achieved through these techniques will pave the way for a transformative era in gastrointestinal (GI) disease modeling and treatment strategies. This review serves as an introduction to the companion protocol paper featured in this journal. The protocol outlines the isolation and co-culture of myenteric and submucosal neurons with small intestinal organoids. This review provides an overview of the intestinal organoid culture field to establish a solid foundation for effective protocol application. Remarkably, the ENS surpasses the number of neurons in the spinal cord. Referred to as the “second brain”, the ENS orchestrates pivotal roles in GI functions, including motility, blood flow, and secretion. The ENS is organized into myenteric and submucosal plexuses. These plexuses house diverse subtypes of neurons. Due to its proximity to the gut musculature and its cell type complexity, there are methodological intricacies in studying the ENS. Diverse approaches such as primary cell cultures, three-dimensional (3D) neurospheres, and induced ENS cells offer diverse insights into the multifaceted functionality of the ENS. The ENS exhibits dynamic interactions with the intestinal epithelium, the muscle layer, and the immune system, influencing epithelial physiology, motility, immune responses, and the microbiome. Neurotransmitters, including acetylcholine (ACh), serotonin (5-HT), and vasoactive intestinal peptide (VIP), play pivotal roles in these intricate interactions. Understanding these dynamics is imperative, as the ENS is implicated in various diseases, ranging from neuropathies to GI disorders and neurodegenerative diseases. The emergence of organoid technology presents an unprecedented opportunity to study ENS interactions within the complex milieu of the small and large intestines. This manuscript underscores the urgent need for standardized protocols and advanced techniques to unravel the complexities of the ENS and its dynamic relationship with the gut ecosystem. The insights gleaned from such endeavors hold the potential to revolutionize GI disease modeling and treatment paradigms.

## 1. Introduction

**Figure 1:**
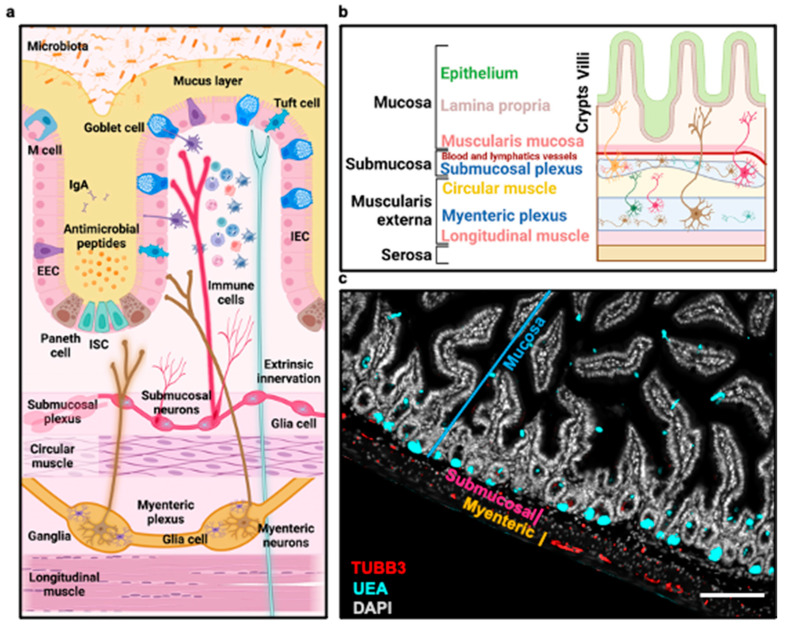
Intricate structure of the enteric nervous system (ENS) within the small intestine. (**a**,**b**) Visual illustration of the small intestinal mucosa comprising the intestinal epithelium organized into villi and crypts. The intestinal epithelium houses various cell types, including intestinal stem cells (ISCs), Paneth cells, enterocytes, goblet cells, tuft cells, and enteroendocrine cells (EEC). The epithelium is supported by a layer of connective tissue known as the lamina propria, containing a diverse array of immune cell types. Additionally, nested within the mucosa resides a layer of smooth muscle known as the muscularis mucosa. Beneath the mucosa lies the submucosa, a thick layer of loose connective tissue that contains blood vessels, lymphatic vessels, and the submucosal plexus. This plexus comprises ganglia containing intraganglionic glial cells and submucosal neuronal bodies, which extend intrinsic innervations toward the mucosa and establish connections with its cellular components. The smooth muscle responsible for facilitating intestinal motility is found within the muscularis externa, which consists of two distinct layers: the inner circular layer and the outer longitudinal layer. Between these layers lies the myenteric plexus. Intrinsic nerves originating from the ganglia of the myenteric plexus extend outward and provide innervation to the cellular components of the small intestine. Separating the muscularis externa from the serosa, there is an outer layer of connective tissue known as the adventitia. Figures created with BioRender.com, (accessed on 2 November 2023). (**c**) Representative section of immunofluorescent staining was conducted on the proximal small intestine. 4′,6-diamidino-2-phenylindole (DAPI) (gray) was used to label the cell nuclei, ulex europaeus agglutinin (UEA) stains goblet cells (cyan), and tubulin beta-III (TUBB3) (red), specifically, stains enteric neurons. Scale bar = 100 μm.

Within the intestinal mucosal barrier, which encompasses the intestinal epithelium, the microbiota, the mucus layer, the enteric nervous system (ENS), and the immune system, a delicate and precisely orchestrated equilibrium known as intestinal homeostasis plays a pivotal role in maintaining health. Together, these components collectively govern the ever-evolving composition of the intestinal microbiota [1]. The ENS is composed of enteric neurons and glial cells, which, together, form two important ganglionated plexuses. These plexuses are known as the myenteric plexus, situated between the longitudinal and circular muscle layers, and the submucosal plexus, located beneath the mucosa (Figure 1a–c). The submucosal plexus exhibits variations across species. The ENS typically consists of a single layer of ganglia in common laboratory animals, such as mice, rats, and guinea pigs. In larger mammals, including humans, it consists of two layers known as the inner and outer submucosal plexus [2]. The myenteric plexus plays a crucial role in controlling intestinal motility, including the rapid orthograde propulsion of contents (peristalsis), mixing movements (segmentation), slow orthograde propulsion (the migrating myoelectric complex), and retropulsion (the expulsion of substances associated with vomiting) in the gastrointestinal (GI) tract [3,4]. The submucosal plexus (or inner submucosal plexus) is responsible for the regulation of functions such as absorption, secretion, and the detection of stimuli [2,5]. In larger animals, a subset of motor neurons in the outer submucosal plexus assumes a significant role in regulating the circular and longitudinal muscles [6,7]. Furthermore, in larger animals, there are scattered enteric neurons at the base of the mucosa, controlling mucosal functions [6,7]. The GI tract features a sophisticated network of ganglia interconnections spanning its entire length, including projections between the myenteric and submucosal plexuses [2]. The communication between the enteric neurons occurs through the release of neurotransmitters from axonal swellings called varicosities [2]. Intrinsic primary afferent neurons (IPANs), also known as intrinsic sensory neurons, play a pivotal role as the main sensors and regulators of the ENS. These neurons form connections with each other and respond to mechanical and chemical signals from the gut lumen. They relay this information to various types of neurons, including secretomotor neurons, vasodilator neurons, interneurons, and motor neurons [2,8]. Mostly located in the myenteric plexus, IPANs extend their processes into the lamina propria beneath the epithelial layer [9]. The interneurons within this plexus then connect with different types of motor neurons, including those that regulate circular and longitudinal smooth muscle contractions, vasomotor neurons, secretomotor neurons, motor neurons, and viscerofugal neurons. The viscerofugal neurons play a role in intestino-intestinal reflexes, which are reflex actions that occur within the intestines themselves. These neurons transmit signals from one part of the intestine to another, aiding in the local coordination of intestinal functions [8,10]. Motor reflexes can be triggered by the stretching or distension of the gut, even without the involvement of mechanosensory elements in the mucosa [8,10]. The ENS innervates the epithelium, smooth muscle, interstitial cells of Cajal (ICCs), vasculature, and immune cells to monitor and respond to alterations. In the epithelium, various types of epithelial cells, including enteroendocrine cells and goblet cells, interact with enteric neurons and glial cells to modulate GI physiology. Therefore, the extensive and complex interconnections of the ENS enable the coordinated regulation of various gut functions, such as secretion and motility [11]. The delicate balance of these intricate systems is disrupted in GI diseases, including liver disease [1,12,13,14,15].

This review complements the companion protocol manuscript outlined in this journal, which details the isolation and co-culture of myenteric and submucosal neurons with small intestinal organoids. Our objective is to provide a foundational understanding of this method, offering background information and discussing its significance and applicability. Recognizing the unique traits and interactions between the ENS and the epithelia of the small or large intestines, the muscle layers, and the intestinal immune system is crucial for advancing the research in this field. The advance of organoid technology holds significant potential for elucidating the complex interplay among various constituents of the intestinal mucosal barrier, including the ENS and the intestinal epithelium. This review emphasizes the urgent need for innovative co-culture systems that integrate the ENS with intestinal organoids, as well as other intestinal mucosal barrier components underlining the importance of developing sophisticated models for a deeper understanding and treatment of GI disorders. The collective efforts of the scientific community in devising and utilizing these models to decipher the unique characteristics, biomarkers, and therapeutic targets of diseases offer promising prospects for revolutionizing our understanding of GI pathologies and formulating novel treatment modalities. Such pioneering advancements are poised to pave the way for more precise, effective, and individualized therapeutic approaches. 

### 1.1. Mucosal Barriers

The integrity of mucosal barriers is of great importance in preserving overall health. An essential constituent of mucosal surfaces is the epithelial cell layer, which comprises various specialized cell types like enterocytes, goblet cells, intestinal microfold cells (M cells), enteroendocrine cells, tuft cells, intestinal stem cells (ISCs), and Paneth cells situated within the crypts [16]. The epithelial cell layer establishes a physical and chemical barrier to the external environment. The physical barrier includes the mucus layer, the glycocalyx on the intestinal epithelial cells (IECs), and the tight junctions between them. The chemical barrier comprises antimicrobial molecules, including immunoglobulin A (IgA) and the defensin family of proteins, as well as the release of inflammatory mediators, such as chemokines and cytokines [16] (Figure 1a).

The intestine varies in composition throughout its length [17]. In the upper small intestine, long, thin villi are covered by a surface epithelium with microvilli containing digestive enzymes that facilitate absorption. As the intestine progresses, the villi become shorter, and the population of goblet and Paneth cells increases. The caecum serves as a reservoir for commensal bacteria, aiding in fermentation during digestion. It lacks villi but contains numerous goblet cells. In the colon, villi are absent, the crypts are smaller, and there are abundant goblet cells but scarce Paneth cells. The surface epithelium primarily reabsorbs water and acts as a barrier to bacteria [17].

Goblet cells secrete mucin to form mucus, while Paneth cells produce antimicrobial peptides [18]. In the small intestine, goblet cells are less abundant than in the large intestine, however, several IECs, including Paneth cells, release antimicrobial peptides contributing to the formation of a chemical barrier aiding in a microbial defense [19]. Notably, the large intestine contains two mucus layers: an inner layer devoid of bacteria and an outer layer, which is larger in volume, less dense, and penetrable to bacteria. Conversely, the small intestine possesses a single, discontinuous mucus layer that is relatively porous, enabling bacterial infiltration [18]. The mucus thickness varies throughout the GI tract and across species. Commensal bacteria and pathogens have developed various strategies to inhabit specific niches within the mucus barrier [18].

The gut microbiome interacts with the mucus layer through various mechanisms. Commensal bacteria utilize strategies such as surface adhesion and the enzymatic degradation of mucin glycans for colonization [20,21,22]. Overall, the intricate relationship between the gut microbiome and the mucus layer is crucial for maintaining gut homeostasis and protecting against infections. Pathogens manipulate mucosal glycosylation to promote colonization [23], while commensal bacteria produce antibacterial compounds and strengthen the mucus barrier [23]. Factors like pH, viscosity, and antimicrobial agents influence mucus function [24,25,26]. Additionally, bacteriophages interact with mucins to protect against dysbiosis [27], although their role is complex and still being studied [28].

Another component of the mucosal barrier is the intestinal immune system. The intestinal immune system plays a pivotal role in regulating the expression of antimicrobial peptides and secretes cytokines and immunoglobulins participating in the antimicrobial response [29]. Immune cells reside in various regions of the gut, including organized structures like gut-associated lymphoid tissue (GALT) and mesenteric lymph nodes, as well as being scattered throughout the epithelium and lamina propria. GALT and draining lymph nodes are crucial for priming adaptive immune responses, while effector immune cells are dispersed across the lamina propria and epithelium [17]. The lamina propria contains a diverse range of immune cells, including B cells, T cells, dendritic cells, macrophages, eosinophils, and mast cells. T cells predominate in the epithelium. Regional differences in immune cell distribution and function exist along the intestine [17]. In the small intestine, the focus of the immune system is on preserving epithelial function. This is achieved by the presence of monitoring IL-17- and IL-22-producing T cells, innate lymphoid cells (ILCs), and intraepithelial T cells with innate and cytolytic and antimicrobial peptides induction function [17,30]. In addition, regulatory T cells (Tregs) help to prevent hypersensitivity reactions to dietary antigens [17,31]. Meanwhile, the large intestine houses a larger and diverse array of beneficial commensal microorganisms crucial for our health. Despite being perceived as potential threats by the immune system, these microorganisms are effectively managed through mechanisms such as the production of a thick mucus layer, IgA antibodies, and regulatory T cells [17,32].

These orchestrated actions of the innate and adaptive immune cells promote immunosurveillance mechanisms that are indispensable for safeguarding the overall defense [33].

Additionally, the intestinal mucosal barrier contains four unique subpopulations of myenteric glia, which are differentially distributed between the colon and the duodenum, contributing to region-specific mechanisms that regulate digestive functions [34]. In the same line, enteric neurons exhibit heterogeneity along the digestive tract [5]. This subject will be elaborated upon later in this manuscript.

The compromised functionality of these barriers, as seen in mucosal inflammatory disorders like inflammatory bowel disease (IBD) and liver diseases, has been extensively documented [1,12,13,14,15]. The global prevalence of GI disorders continues to escalate [35], making it imperative to gain an understanding of the initiation and pathophysiology of these conditions.

### 1.2. Culturing Techniques: Intestinal Organoids and Their Applications

Prior to the isolation of ISCs, the scientific community relied solely on intestinal tumor cells as the model for studying intestinal cells. The frequently utilized Caco-2, HT-29, and T84 cell lines were obtained from human colorectal adenocarcinoma [36,37,38]. The culture of intestinal organoids, as a remarkable innovative three-dimensional (3D) technique mimicking the crypt-villus unit, has advanced our understanding and provided insights into the complexities of the intestinal system [39,40,41,42]. The term “organoid” is used extensively for ex vivo cultures, but it necessitates further clarification to distinguish between the different types. Organoids can be derived from multipotent ISC from isolated crypts of Lieberkühn, obtained from the small intestine (enteroids) or the colon (colonoids). Alternatively, organoids can be grown from embryonic stem cells (ESC) from blastocysts. Other sources are those obtained from induced human pluripotent stem cells (iPSCs) to create human intestinal organoids (HIO) [39,40,41,42,43]. In the initial stages of the culture process, a specific type of cellular structure is created (spheroids), formed by growing and maintaining ISC. ISCs, such as crypt base columnar cells (CBCs), are leucine-rich repeat-containing G protein-coupled receptor 5 (LGR5+) and divide rapidly and produce transit-amplifying (TA) cells, which in turn differentiate into absorptive (enterocyte) or secretory (Paneth, goblet, and enteroendocrine) cell fates [39,44]. These intestinal organoids are cultivated with factors indispensable for stem-cell maintenance, including wingless-type mammary tumor virus (MMTV) integration site family member 3A (Wnt3a), epidermal growth factor (EGF), noggin (a bone morphogenetic protein (BMP) inhibitor), and R-spondin 1 (a ligand of LGR5 and WNT agonist), which are crucial for their growth, mimicking the in vivo conditions [45,46,47]. Cultivated with these components, intestinal organoids maintain their integrity and display a distinctive polarity, where the apical sides are oriented towards the internal lumens, while the basolateral domains are in contact with the complex extracellular matrix (ECM) gel and the surrounding media.

This culture method is versatile and applicable for cultivating both mouse and human organoids, allowing for continuous growth over extended periods of time. Due to their ability to mimic the features of the original intestinal tissue, such as gene expression, polarization, nutrient and ion transport, barrier function, mucus secretion, antimicrobial peptide production, cytokine and chemokine expression, organization, and the fact that they contain progenitor and differentiated cells, they are a valuable tool for understanding physiological and pathological processes. Indeed, the growth of 3D intestinal organoids derived from isolated intestinal crypts from mouse or human origin [39,40,41,45,48] and the growth of HIOs derived from iPSCs differentiated into definitive endoderm, then into mid/hindgut tube spheroids, and finally into organoids [41,49] is a significant advance in the field. For instance, intestinal organoids have revolutionized various fields, including drug discovery, genetic profiling, the study of intestinal transporters, and host–pathogen interactions [50,51,52,53,54,55,56,57]. They have proven valuable in advancing our understanding of the onset of GI diseases and have greatly enhanced our comprehension of intestinal biology [58]. Moreover, advances in gene editing techniques, such as Clustered Regularly Interspaced Short Palindromic Repeats-Cas9 (CRISPR)-Cas9, have enabled the induction of specific somatic mutations in organoids, opening doors to modeling intestinal diseases and cancer [59,60,61,62].

Undoubtedly, the ongoing advances and applications of organoid technology are driving breakthroughs. The latest advances in organoid culture involve scaffold-free and scaffold-based systems. In the scaffold-free system, cells aggregate to form microtissue spheroids using hanging drops, magnetic fields, or specialized synthetic materials [38,63,64,65]. In the scaffold-based system, the cells attach to scaffolds composed of natural ECM [66] or synthetic materials, including hydrogels [67] or solid porous structures [68]. Each type of scaffold has its advantages and limitations, offering researchers a variety of options for 3D cell culture based on their specific research needs. There are other notable progressions, such as the use of collagen in place of Matrigel. This collagen-based approach induces a phenomenon termed “fetalization,” a process that involves partial adoption of the fetal intestine-specific phenotype by intestinal organoids derived from adults. This fetalization process has been observed in patients with ulcerative colitis (UC), rendering it of significant clinical relevance [69,70].

In addition to the aforementioned advances, ex vivo cultured ISCs can engraft and contribute to the regeneration of damaged mucosa in the colon, offering potential therapeutic implications for refractory IBD [69,71,72]. Building upon this knowledge, researchers have developed an induced human UC-derived organoid (iHUCO) model using iPSCs to better understand UC, a type of IBD. iHUCOs exhibit histological and functional features similar to primary colitic tissues, including aberrant epithelial barrier characteristics. The model also revealed an overexpression of the C-X-C motif chemokine ligand 8 (CXCL8)–C-X-C chemokine receptor type 1 (CXCR1) axis, which was not observed in the induced human normal organoid model (iHNO). CXCL8 is one of the most important proinflammatory factors that play a vital role in many inflammatory diseases, including UC [73]. The CXCL8–CXCR1/2 axis participates in the pathogenesis of UC through multiple signaling pathways, including phosphoinositide 3-kinase (PI3K)/protein kinase B (Akt), mitogen-activated protein kinase (MAPKs), and nuclear factor kappa-light-chain-enhancer of activated B cells (NF-κB) signaling pathways [73]. Using iHUCOs, it has been demonstrated that the overexpression of CXCL8–CXCR1 in UC results in a dysregulated adherens junction pattern in epithelial cells. Notably, CXCL8 lacks a murine homolog, highlighting the gap in murine-based models and the functional importance of human-based models. The functionality of the model was demonstrated via the response to chemical perturbation by repertaxin, a CXCR1 receptor small molecule non-competitive inhibitor. Repertaxin attenuated several aspects of the colitic phenotype, including a leaky epithelial barrier. These results suggest that the pro-inflammatory interaction of CXCR1–CXCL8 compromises the epithelial barrier. Additionally, UC patient tissues overexpress CXCL8 and its receptor. Therefore, using repertaxin to block this interaction may be a promising therapeutic strategy to diminish the chronic inflammatory symptoms of UC. Indeed, inhibiting the CXCL8 receptor with repertaxin attenuated UC phenotypes both in vitro and in vivo, showcasing the potential for tailored interventions using this patient-derived organoid model containing epithelial and stromal compartments [74].

Intestinal organoid models hold promising potential in biomedicine. However, they are encumbered by notable limitations. Among them, the absence of a vascular system, smooth muscle, associated ICCs, connective tissue containing fibroblast, a nervous system, and immune systems represents a significant drawback [75]. Researchers have been exploring ways to enhance the functionality and complexity of intestinal organoids by co-culturing them with specific cell types [76,77,78,79,80,81]. Introducing intestinal subepithelial myofibroblasts (ISEMFs) has led to the long-term culture and growth of organoids [82], and co-culture with vagal neural crest cells (NCCs) has enabled the development of functional neurons and glia, simulating the ENS [83]. Furthermore, co-culturing with immune cells and providing interleukins has been shown to improve organoid growth and maturation [84]. Attempts have been made to incorporate blood vessels into organoids to mimic the in vivo intestinal environment [53].

Additionally, the co-culture of GI organoids with microbiota holds particular relevance, as the intestinal microbiota influence various aspects of intestinal biology, including epithelial turnover, physiological processes, immune homeostasis, and drug pharmacokinetics. Intestinal organoids have also been used to study the interactions between the intestinal epithelium and various bacteria, including probiotic and pathogenic strains. They have shown that probiotic *Lactobacillus* species enhance organoid growth and maturation [85], while pathogenic bacteria like *Escherichia coli* and *Salmonella* can cause damage to the intestinal epithelium [86,87]. Microinjecting pathogens such as *Clostridium difficile* or *Cryptosporidium parvum* into the lumen of organoids provides excellent examples of established infection models [88,89]. Additionally, organoids have been instrumental in studying the replication and effects of enteric viruses like rotavirus and norovirus, providing valuable insights into GI diseases and potential therapies [90,91,92]. Overall, intestinal organoids offer a powerful tool to investigate microbiota interactions and understand GI tract diseases, paving the way for improved treatments and preventive measures.

Current developments focus on overcoming limitations by employing methods such as 3D bioprinting, biomaterials, and co-culture [93,94]. Bioprinting and microfluidic devices are cutting-edge techniques that enable the encapsulation of various cells in compatible hydrogels and precise placement for co-culture [95,96,97]. These methods allow researchers to create tissues with intricate vascular networks and innervation, enhancing tissue complexity. Regarding bioprinting, research has demonstrated that “assembloids” created through cell-based 3D printing technology surpass organoids and exhibit structure and function that closely resemble human tissues and organs [98]. Microfluidic devices, in particular, facilitate the spatial separation of different cell types, making them ideal for modeling complex interactions, such as angiogenesis, by connecting organoids and vascular cells through soluble factors [99]. The integration of microfluidic devices with organoids is termed “organoid-on-a-chip” [100]. The concept of organoids-on-a-chip represents a more intricate cultivation approach, where the autonomous arrangement of stem cells can be externally influenced through a 3D microstructured scaffold. This microarray technology enables the regeneration of functional intestinal microarchitecture with physiologically relevant shear stress and mechanical motions, as well as the introduction of anaerobic bacteria [54]. It has the potential to advance the field by facilitating the study of different cell types and intestinal epithelium–microbiota interactions [101]. Furthermore, ongoing research focuses on engineering materials to use as matrices for organoid culture to improve organoid development and functionality [102,103]. Other advances include the generation of organoids with inverted polarization, with the apical side facing the ECM [104]. Other advances include promoting their growth on monolayers [105,106,107], the utilization of microinjection techniques to study host–microbiome interactions [77], and promoting specific differentiation to different cell types [108,109,110,111]

Despite the impressive complexity of organoids, they still fall short of containing all of the necessary cell and tissue types essential for achieving full organ function, such as the ENS. Nevertheless, pioneering studies have harnessed engineered organoid systems to shed light on how surrounding cells and microbiota influence GI pathophysiology by establishing co-cultures of organoids with microbes, immune cells, neural cells, or stromal cells, among others [76,77,78,79,80,81]. Yet, in order to truly propel the field forward, further advances are needed. The current techniques, involving multiple co-cultures and strategies for reconstituting culture environments, demand refinement to elevate both the structural intricacy and the functional capabilities of these systems. By addressing these challenges, we can unlock new frontiers in regenerative medicine and drive the development of cutting-edge treatments and therapies. The quest to achieve organoids that closely mimic the complexity and function of human organs holds immense promise for revolutionizing biomedical research and healthcare.

### 1.3. The Enteric Nervous System (ENS)

Within the digestive tract, the ENS assumes a pivotal role, functioning as an intrinsic neuronal network that governs the GI functions alongside the extrinsic innervation provided by the parasympathetic and sympathetic components of the autonomic nervous system [112]. The ENS consists of a vast array of neurons with diverse functions and glial cells. These elements are organized into the myenteric and submucosal plexuses. Notably, their number surpasses those in the spinal cord (Figure 1a–c). The complexity of this system escalates when considering that the neurochemical profiles are influenced by factors such as the gut environment, endocrine influences, and interactions with the microbiota [113,114,115]. Immunohistochemistry, morphological, and single-cell RNA sequencing (scRNAseq) analyses have been instrumental in the precise classification of enteric neuron subtypes. Through these techniques, studies have elucidated the primary neurotransmitters that delineate various neuron types within the myenteric plexus. While most myenteric neurons are traditionally classified as either cholinergic or nitrergic, research has unveiled complexities, with some neurons exhibiting dual characteristics, lacking both, or expressing glial markers. These facts challenge the established paradigms and underscore the criticality of accurate classification when exploring enteric neuronal populations [116,117,118]. Submucosal neurons, on the other hand, mainly consist of cholinergic neurons and vasoactive intestinal peptide (VIP)–expressing noncholinergic neurons. These major neuronal populations can further divide into subsets based on additional markers like neuropeptides and calcium-binding proteins [4]. The proportions of these subsets vary along the gut and show interspecies differences. Overall, around 15 classes of functionally defined, neurochemically coded enteric neurons have been identified in the intestine, with fewer in the stomach and esophagus [2].

In 2008, Qu et al. characterized nerve cell types in the mouse small intestine’s myenteric plexus using antibodies to define the neurons by shape, size, and neurochemistry. They found type II neurons, representing 26% of the neurons, with axons projecting to the mucosa and expressing choline acetyltransferase (CHAT) and vesicular acetylcholine transporter (VACHT). It was also described that nitric oxide synthase (NOS) occurred in 29% of neurons, mostly inhibitory motor neurons to the muscle. Calretinin (CR) was found in 52% of neurons, with some identified as excitatory neurons. Overall, this work defined that over 90% of all neurons can be identified by their neurochemistry and shape, aiding in understanding their function [119].

Subsequent studies by Foong et al. in 2014 focused on the submucosal neurons in the mouse distal colon, shedding light on their role in regulating gut secretion and elucidating regional differences in neurochemistry and ion transport responses. Using (ChAT)-Cre × ROSA26^YFP^ reporter mice, which express a yellow fluorescent protein (YFP) in neurons that express CHAT, they correlated the neurochemistry, morphology, and electrophysiology of submucosal neurons. They identified two main neurochemical groups: cholinergic and non-cholinergic neurons, with the majority in the distal colon being non-cholinergic but containing VIP. They found that the distal colon had smaller ganglia, a higher proportion of cholinergic neurons, and a larger nicotinic secretory component compared to the proximal colon. Their study highlights the regional differences in submucosal neurons and underscores the need for further investigation [120].

In 2020, Morarach et al. identified 12 enteric neuron classes within the myenteric plexus of the mouse small intestine using scRNAseq. Together with transgenic tools for class-specific targeting, this group elucidated cell–cell communication features and histochemical markers of motor neurons, sensory neurons, and interneurons, [121].

Similarly, Drokhlyansky et al., in 2020, provided a molecular characterization of the ENS in adult mice and humans at single-cell resolution, uncovering extraordinary neuron diversity and identifying conserved and species-specific transcriptional programs. They developed two innovative methods, ribosomes and intact single nucleus (RAISIN) RNA-seq and mining rare cells sequencing (MIRACL)-seq, to profile the ENS with unprecedented detail and resolution. By applying these techniques, they generated an atlas of the adult ENS spanning species, age, sex, region, and circadian phase. In their mouse atlas, which includes data from the ileum and colon, they identified a great diversity of neurons, comprising 21 distinct neuron subsets and 3 glia subsets. Notably, they observed circadian expression changes in the enteric neurons and found a dysregulation of disease-related genes with aging. Differences between the ileum and colon were also identified, indicating regional variations in gene expression and neuron proportions. Similarly, in their human atlas, the researchers profiled over 400,000 nuclei and identified 1445 neurons, revealing conserved and species-specific transcriptional programs. They uncovered putative neuro–epithelial, neuro–stromal, and neuro–immune interactions, indicating the complex interplay within the ENS. Importantly, they found that the human ENS expresses genes associated with neuropathic, inflammatory, and extra-intestinal diseases, suggesting potential neuronal contributions to disease pathogenesis. Moreover, their study provided valuable insights into age-related changes, regional differences along the intestine, and circadian regulation of the ENS. By comparing the gene expression between humans and mice, they identified conserved transcriptional programs across species, while also highlighting differences in key pathways. The study also inferred putative interactions between the ENS and various cell types, shedding light on the role of the ENS in mucosal immunity and disease pathogenesis [122].

In 2021, Wright et al. aimed to better understand the molecular landscape of enteric neuron subtypes to aid in the development of therapeutic strategies for enteric neuropathies and to enhance our knowledge of ENS function. They conducted single-nucleus RNAseq (snRNAseq) on adult mouse and human colon myenteric plexuses, as well as scRNAseq on E17.5 mouse ENS cells. Their analysis revealed seven adult neuron subtypes and eight E17.5 neuron subtypes, along with hundreds of differentially expressed genes. Furthermore, the RNAseq data from the manually dissected human colon myenteric plexus provided valuable insights into the gene expression profiles of various cell types within the ENS and surrounding tissues. Immunohistochemistry confirmed the differential expression of several genes, including zinc finger protein basonuclin-2 (BNC2), PBX homeobox 3 (PBX3), SATB homeobox 1 (SATB1), RNA biding fox-1 homolog 1 (RBFOX1), T-box transcription factor 2 (TBX2), and TBX3, in enteric neuron subtypes. Overall, these findings provide valuable insights into the molecular landscape of the myenteric neuron subtypes [123]. This knowledge not only facilitates molecular diagnostic studies, but also holds promise for the development of novel therapeutics targeting bowel motility disorders.

In 2022, May-Zhang et al. identified a total of 10 enteric neuron subtypes in the duodenum, 13 in the ileum, and 14 in the colon in mice. This group conducted a study comparing the types of enteric neurons found in the small intestine and colon of humans and mice. By analyzing the genetic profiles of these neurons, they discovered similarities and differences between species. Some enteric neuron subtype-specific genes found in mice were expressed differently in humans, indicating distinct neuron subtypes. These findings suggest that caution is needed when making cross-species inferences for specific EN subtypes. Examining multiple regions of the GI tract with snRNAseq, this group identified 22 myenteric EN subtypes throughout the entire intestine. This study also revealed regional variations in gene expression along the GI tract. For example, the expression of certain genes differed between the small intestine and the colon in both humans and mice. This regional variation suggests the potential for developing targeted therapies for specific enteric neuron subtypes in different parts of the intestine. This research expands our understanding of enteric neuron diversity and provides insights into potential treatments for GI disorders. Additionally, this study identified enteric neuron genes that are differentially expressed between males and females, offering new avenues for investigating sex-related differences in motility disorders. Overall, this comparative molecular analysis enhances our knowledge of enteric neuron subtypes across species and intestinal regions, providing a foundation for diagnosing enteric neuropathies and other GI diseases with a neuronal basis [124].

Furthermore, scRNAseq studies, complemented by earlier double- and triple-label immunofluorescence investigations, have facilitated the integration of transcriptome data with pre-existing functional identifications. These studies revealed that neurochemically distinct classes of enteric neurons express multiple mediators in various combinations. For instance, VIP and neural NOS (nNOS) coexist in inhibitory motor neurons, certain interneurons, and a subset of submucosal VIP neurons, demonstrating the intricate nature of neurochemical co-localization [116,117,118,121,125]. Therefore, caution is warranted, due to limitations in immunohistochemistry, especially the use of restricted markers such as CHAT, nNOS, or VIP to define specific subtypes. Recent studies have identified similarities between humans and rodents among some EN subtypes, but caution is advised regarding species differences when translating research from non-conserved EN morphology [124].

In the ENS, neuronal cell bodies are clustered into groups known as ganglia, surrounded by glia, with neuronal axons projecting connections among other ganglia and the epithelium [112]. Glial cells support the homeostatic function of intestinal neurons, forming a communication network and regulating immunity and cell growth [126]. Remarkably, enteric neurons within distinct plexuses exhibit diverse functions and comprise numerous subtypes, including IPANs, interneurons, and motor neurons [2]. Enteric motor neurons drive intestinal motility.

Sensory neurons receive various sensory stimuli from the mucosa and muscle [2]. Two categories of intrinsic IPANs have been described: myenteric and submucosal IPANs. Myenteric IPANs are responsive to distortions in their processes within the outer muscle layers and to changes in the luminal chemistry through extensions into the mucosa. Submucosal IPANs detect any mechanical deformation of the mucosa and shifts in the luminal chemistry. However, there is little direct evidence regarding the sensory modalities served by submucosal IPANs. Conversely, extrinsic primary afferent neurons originate from cell bodies situated in dorsal root ganglia (referred to as spinal primary afferent neurons) and in vagal ganglia (encompassing nodose and jugular ganglia). Despite scRNAseq technologies being instrumental in identifying potential groupings of enteric neurons, their ability to directly define functional subclasses of IPANs is limited. Furthermore, scRNAseq studies predominantly focus on myenteric neurons, with little emphasis on the submucosal plexuses. While physiological evidence suggests the presence of different types of IPANs, a systematic relationship between scRNAseq findings and physiological evidence has not yet been established [2,5,127,128,129,130].

In the small and large intestine, uniaxonal interneurons play a vital role. They used to be categorized into two types: either ascending (oral projection, targeting excitatory motor neurons) or descending (anal projection, targeting inhibitory motor neurons). They are distributed differently in the small and large intestine. For instance, in the ileum of the guinea pig, there is a single class of excitatory ascending interneurons alongside three classes of descending interneurons. Conversely, in the colon, the composition differs, with three classes of ascending interneurons and four classes of descending interneurons. Their distribution varies between the small and large intestine [125,131]. However, the traditional view of a linear ascending-to-excitatory and descending-to-inhibitory neural pathway is outdated. New evidence suggests that interneurons form complex connections, including cross-connections between excitatory and inhibitory pathways. Ascending and descending interneurons form extensive synaptic connections with one another, enabling mutual activation. These findings have shown temporal coordination in the firing patterns of large populations of excitatory and inhibitory motor neurons during aboral fluid propulsion. These neurons act both orally and aborally to facilitate the propagation of contractions in colonic smooth muscle [132]. Interneurons, categorized by neurochemical classes, primarily employ acetylcholine (ACh) as the primary neurotransmitter; however, each subtype may utilize other co-transmitters, such as 5-hydroxytryptamine (5-HT), adenosine triphosphate (ATP), tachykinin (TK), nitric oxide (NO), and somatostatin (SOM) [133].

In the myenteric plexus, excitatory motor neurons use ACh to contract circular and longitudinal muscles [133]. Inhibitory neurons employ various co-transmitters like NO, ATP, β-nicotinamide adenine dinucleotide (β-NAD), VIP, and pituitary adenylate cyclase-activating polypeptide (PACAP) [128,133,134]. It has been reported that the release of neurogenic purines contributes to tonic inhibition in the colon [135]. Historically, ATP has been considered the primary purine neurotransmitter [136,137,138,139]. However, recent investigations involving mouse and primate colons have revealed that another purine, β-NAD+, along with its bioactive derivative, adenosine 5′-diphosphate ribose (ADPR), may more effectively replicate the actions of the endogenous purine neurotransmitter compared to ATP [140,141,142]. In the GI tract, alongside myogenic control, various hierarchical regulatory systems orchestrate coordinated muscular movements for normal GI motility. Smooth muscle cells are interconnected via gap junctions with two key types of interstitial cells: ICCs and platelet-derived growth factor receptor α positive (PDGFRα+) cells. Together, these form an electrical syncytium termed the smooth muscle cells/ICCs/PDGFRα+ (SIP) syncytium [143,144]. Inward and outward conductances within these cells influence the overall muscle excitability and responses to regulatory inputs. ICCs act as pacemaker cells and integrate inputs from motor neurons [145,146], while PDGFRα+ cells likely mediate the purinergic inputs from the enteric inhibitory motor neurons [147]. PDGFRα+ cells express the necessary molecular machinery for transducing these inputs, generating spontaneous Ca^2+^ transients and dynamic Ca^2+^ oscillations in response to purines [148]. Purinergic responses involve P2Y1 receptors and Ca^2+^ release from intracellular stores [148]. Ca^2+^ release in PDGFRα+ cells activates Ca2^+^-activated K+ channels, leading to hyperpolarization in GI muscles and eliciting inhibitory motor responses [148]. Spontaneous Ca^2+^ transients may regulate the basal excitability of fundus muscles, and purine release from motor neurons may aid in controlling pressure during stomach filling in the proximal stomach [148]. Motor pattern generators are pivotal in orchestrating peristaltic movements for content propulsion, as well as segmentation to optimize digestion, and are tightly regulated for coordination with secretion.

When IPANs detect luminal stimuli, they excite ascending interneurons, initiating ascending excitation. This activation leads to the stimulation of excitatory motor neurons, inducing oral contractions. Meanwhile, descending interneurons are activated to stimulate inhibitory motor neurons, facilitating anal relaxation to allow easier movement of contents to propel them [149]. It is important to note that descending excitation is often seen, especially in the colon, and overall anterograde propulsion requires anally propagating contractions [150,151].

In addition, enteric glia cells [152] and luminal microbiota influence colonic motility and the transcription profile of enteric neurons. This occurs via the activation of aryl hydrocarbon receptor (AhR) signaling [153]. Furthermore, macrophages in the muscularis externa modulate peristalsis via BMP2 secretion, interacting dynamically with enteric neurons influenced by microbiota signals [154].

In the submucosal plexus, the secretomotor and vasodilator neurons regulate intestinal secretion and blood flow in the mucosa and submucosal vasculature. While both populations employ ACh and VIP as key transmitters, they also express additional co-transmitters [128]. Chemical and mechanical stimulation of the mucosa excites myenteric or submucosal sensory neurons. These, in turn, activate the secretomotor and vasodilator neurons directly or via interneurons. Additionally, they may directly regulate secretion through an axon reflex [128]. Through the transmission of excitatory or inhibitory signals via neurotransmitters, the enteric neurons orchestrate fluid secretion and absorption, GI motility, blood flow, inflammation, and pain perception [5]. Any alterations in the functioning of these neuronal cells can result in intestinal motor dysfunctions or disrupt the intricate coordination of the intestinal epithelium [155].

### 1.4. Culturing Techniques: ENS

Exploring enteric neurons has long been methodologically challenging, due to the close proximity of the ENS to the contractile gut musculature and its intricate association with other intestinal cell types, including epithelial, immune, and stromal cells, as well as the nonsterile environment within the intestinal lumen [156]. While guinea pigs have historically served as a valuable model for enteric neuronal cultures, their limitations in terms of genetic modification and cost have led to the emergence of murine models [157,158]. Additionally, the culture of enteric neurons from rats and humans has also been established, expanding the scope of research possibilities in this field [159,160]. The evolution of enteric neuron culturing techniques includes primary cell cultures [156], stem cell-derived 3D neurospheres [158], induced ENS cells from stem cell origin [80,161,162], and established cell lines [163]. Enteric neural progenitors, found in the gut’s myenteric and submucous plexus, can be isolated using various methods, including dissection, specialized culture conditions, or cell sorting [164,165,166]. Techniques developed in the late 1970s, such as myenteric and submucosal plexus isolation, initially faced challenges in purity [167,168]. Current approaches, starting with enzymatic dissociation using dispase and collagenase, yield ENS-enriched cultures, but include other cells like fibroblasts and immune cells [165,166]. Cell sorting enhances the purity, though identifying specific markers remains a challenge. Obtaining sufficient ENS cells, especially postnatally, remains a hurdle. Recent protocols using purified collagenase show promise for larger quantities of pure myenteric plexus from the human gut; however, challenges persist in submucosal plexus isolation and understanding differences in stem cell populations [169].

It is important to acknowledge that submucosal and myenteric neurons and glial cells exhibit distinct properties. Depending on the priority, the isolation of different plexuses could be performed and has been already established [170,171,172,173,174,175]. However, when focusing on one plexus alone, the neurons cannot fully reflect the full complexity of the system. Therefore, the choice of the plexus source depends on the scientific objective.

By harnessing the power of in vitro neuronal models, researchers can delve deeper into the intricacies of the ENS. These models shed light on the normal physiology of the ENS and help to unveil the mechanisms underlying various pathological conditions. However, it is imperative to acknowledge that the intricate functionality of the ENS should not be examined in isolation. Given its multifaceted nature, there is a requirement for sophisticated systems that facilitate the study of the dynamic interactions between the ENS and the intestinal epithelium. Although the isolation of the submucosal plexus is possible [172,176], the majority of the studies focus on the isolation of the myenteric plexus for neuronal culture [170,171,172,173,174,175].

### 1.5. ENS, Intestinal Epithelium, and Immune Interactions and Applications

The influence of the ENS on the intestinal epithelium and the intestinal immune system and vice versa has been previously investigated in vivo and ex vivo [8,177,178,179]. Co-culture of HIOs and differentiated ENS from human ESCs resulted in increased epithelial proliferation [80]. Indeed, it has been reported that the ENS can induce genes related to GI development, including EGF and transforming growth factor beta (TGFβ). Additionally, it decreases genes related to goblet and Paneth cell differentiation, while increasing genes related to tuft and enteroendocrine cells. This demonstrates that the ENS regulates epithelial physiology. These co-cultures utilized human-PSC-derived NCCs or primary neurons isolated from the spinal cord [80,180]. However, conflicting results were observed in different co-culture experiments, suggesting variations due to cell sources, developmental age, and, most likely, preparation and culture conditions [162,181,182]. Further instances demonstrate the interconnection between enterochromaffin cells and sensory neural pathways [183]. Regarding the functionality of neurotransmitters, previous studies have demonstrated that 5-HT and ACh release increase epithelial and crypt proliferation indexes [180,184]. Conversely, proliferation decreases with vagotomy or norepinephrine [185], and whole-animal knockouts of muscarinic ACh receptors (M2, M3, and M5) show an increase in epithelial proliferation [186]. Standardized protocols are needed to obtain better comparisons between studies. Enteric glial cells also play a supportive role for IECs. However, conflicting reports exist, highlighting the need for further investigation [187,188,189].

The intestinal epithelium interacts with various components, most notably, the ENS. Understanding neuroimmune–microbiome modulations holds immense promise for the discovery of innovative therapeutic approaches across a spectrum of conditions. Recent advances have shed light on the profound significance of neuronal signals in regulating crucial aspects such as mucosal immune cells, the microbiome, and the integrity of the intestinal barrier [190,191,192,193,194,195]. Notably, unidentified subtypes of VIP-expressing enteric neurons are responsible for directing gut mucosal fucosylation through extracellular signal-regulated kinase 1 and 2 (Erk1/2)-c-Fos pathway. When the abundance of these VIP-expressing neurons decreases, it disrupts the balance between beneficial *Bifidobacterium* and pathogenic *Enterococcus faecalis* in the gut, leading to an increased susceptibility to alcohol-associated liver disease (ALD) [196]. Moreover, the ENS assumes a vital role in orchestrating the goblet cell-derived-antimicrobial peptides response, acting as a potent mediator through the secretion of interleukin (IL)-18. The intricate interplay of the ENS-derived IL-18 and its immunomodulatory effects have been brought to light, culminating in a remarkable defense against pathogens. When ENS-derived IL-18 is deleted in mice, it renders them more susceptible to *Salmonella typhimurium* infection, unveiling the indispensability of this neural network in fortifying the body’s defenses. Importantly, the goblet cell-derived-antimicrobial peptides angiopoietin-4 (*Ang4)*, resistin-like molecule beta *(Retnlb)*, and intelecitin-1 (*Itln1)* were among the most significantly reduced genes in the mice [195].

Research has shown that the intestinal microbiome exerts a profound influence on the ENS [190,197,198,199]. As an illustration, the gut’s colonization by bacteria influences the production of mucosal serotonin and impacts the maturation of the adult ENS [198]. Similarly, the presence of the microbiota exerts a significant impact on neuronal nNOS expression, as evidenced by decreased nNOS+ neurons in the myenteric plexus of germ-free mice, antibiotic-treated mice, and mice with genetic ablation of toll-like receptors (TLRs) [200,201,202]. Notably, enteric neurons express a diverse range of receptors for microbial products, including TLR2, TLR4, and TLR9, whereby genetic ablation leads to alterations in the structure of the ENS [201,203,204]. Additionally, enteric neurons express receptors for metabolites, such as short-chain fatty acids, including the free fatty acid receptor (FFA)-2, FFA-3 [205], and bile acids like G-protein coupled bile acid receptor 5 (TGR5) [206]. The TGR5 receptor plays a crucial role in mediating prokinetic actions of intestinal bile acids and is essential for normal defecation.

As previously mentioned, the ENS innervates and intricately intertwines with the cellular constituents of the intestinal epithelium, thereby influencing their functions [207,208]. Within this array of cellular components, the goblet cells express specific receptors that recognize neurotransmitter ACh, such as muscarinic ACh receptor 4 (M4). Upon engagement with ACh to M4, the goblet cells orchestrate a sophisticated transcytosis mechanism, initiating the formation of goblet cell-associated antigen passages (GAPs). This intricate transcytosis process facilitates the sampling of luminal antigens and bacteria by subjacent antigen-presenting cells (APC). Therefore, this orchestrated interplay positions goblet cells as notable regulators of the intestinal immune system [207,209]. ACh not only regulates goblet cells, but also affects non-neuronal functions like intestinal epithelial ion transport [210]. Endogenous ACh from the intestinal epithelium is essential for maintaining homeostasis and inhibiting differentiation of Lgr5+ ISCs via specific muscarinic ACh receptors (M1, M2, and M3) [211]. ACh can also signal through α2β4 nAChR in Paneth cells, modulating non-canonical Wnt ligands (Wnt5a and Wnt9b) in intestinal organoids. This activates Wnt signaling through Frizzled receptors, promoting enhanced proliferation and differentiation in the stem cell niche [212].

However, neural projections can also reach out to clusters of immune cells, such as C-C chemokine receptor type 6 (CCR6+), type 3 innate lymphoid cells (ILC3s) located in cryptopatches, and isolated lymphoid follicles. Remarkably, this particular subset of ILC3s distinctly expresses VIP receptor type 2 (VIPR2), a receptor for neurotransmitter/neuropeptide VIP. Activated by the intake of food, VIPergic neurons engage with these ILC3s. The interaction of VIP with VIPR2 on CCR6 + ILC3s leads to the inhibition of IL-22 production, a pivotal immune mediator. IL-22, normally elevated by commensal microbes, like segmented filamentous bacteria (SFB), experiences suppression when VIPR2 is engaged. Consequently, this results in a decrease in the production of antimicrobial peptides derived from epithelial cells while simultaneously elevating the expression of lipid-binding proteins and transporters. As a direct outcome of food consumption, the activation of VIPergic neurons plays a pivotal role in fostering the growth of epithelial-associated SFB and amplifying lipid absorption. In essence, these findings uncover a sophisticated dynamic intestinal neuro-immune circuit regulated by feeding and circadian rhythms [213]. In the same line, IL-6 produced by enteric neurons affects the population of microbe-responsive Treg cells in the gut. The immune system and the ENS collaborate to monitor interactions with microbes in the colon. This study described that commensal microbe colonization reduces colon neuronal density, and IL-6 deletion in the neurons increases the Treg cell numbers while reducing the retinoic acid receptor-related orphan receptor gamma (RORγ+) subset. This suggests a circuit where microbial signals impact neuronal activity, influencing Treg cell generation and immune tolerance in the gut [214].

Neuroimmune interactions also play a crucial role during infection. Changes in GI function can aid in clearing pathogens through diarrheal responses that flush out the pathogen by increasing water secretion into the intestine and promoting contractions, both of which are regulated by the ENS [215]. Infections can permanently damage the ENS, leading to disruptions in gut motility and function, resulting in post-infectious GI disorders such as irritable bowel syndrome (IBS) and IBD [195,216].

Understanding the ENS is imperative to unravel its involvement in a wide spectrum of neuropathies. These include congenital conditions like Hirschsprung’s disease, where the ENS is missing from the large intestine. Other conditions include acquired disorders, such as Chagas disease, caused by the parasite *Trypanosoma cruzi*, transmitted to animals and people by insect vectors. Additionally, neuropathies can arise as secondary manifestations from disease states like diabetic gastroparesis, drug-induced complications, and consequences of injuries like postoperative ileus, as well as other GI diseases and conditions involving gut-peripheral organ axes such as gut–liver disease [217,218,219]. Moreover, given that enteric neurons display analogous cellular alterations prior to their manifestation in central neurons in neurodegenerative diseases such as Alzheimer’s disease and Parkinson’s disease, the ENS is a valuable tool for studying the pathogenic process [220,221]. The causes of enteric neuropathies are poorly understood. The interconnectedness between the intestinal epithelium, the ENS, the intestinal immune system, and the microbiota during GI diseases (including liver disease) is altered [2,218]. Therefore, a thorough comprehension of the ENS and its intricate interaction with the GI mucosa is crucial for understanding disease states. The development of systems enabling the study of these interactions is imperative to identify therapies for combating these diseases.

In conclusion, the intricate interplay between the ENS, the intestinal epithelium, the intestinal immune system, and the microbiome necessitates thorough investigation. While significant progress has been achieved in comprehending how the ENS impacts the intestinal epithelium and its role in immune response modulation, challenges and discrepancies persist, due to variations in experimental setups and cell sources. The pursuit of unraveling the precise mechanisms governing these interactions necessitates standardized protocols and innovative techniques to bridge the gaps in our understanding. As we endeavor to unveil the mysteries of the ENS and its dynamic relationship with the gut ecosystem, the development of novel methodologies will play a pivotal role in making breakthroughs that carry immense potential for revolutionizing disease prevention and treatment.

### 1.6. The Imperative of Novel Co-Culture Techniques

The advance of organoid technology holds immense potential for unraveling the intricate interactions between various components of the intestinal mucosal barrier, including the ENS and the intestinal epithelium microenvironment. In comparison to traditional two-dimensional cell cultures, organoid cultures have surpassed expectations in terms of accuracy and physiological relevance, providing invaluable insights into intestinal biology. In a novel in vitro transwell-based co-culture setup, Puzan and colleagues demonstrated that the ENS plays a role in regulating the fate of ISCs. Specifically, the presence of enteric neurons led to an increase in chromogranin A-positive epithelial cells, indicating the promotion of differentiation towards enteroendocrine cells [162]. Recent developments in HIO techniques have significantly enhanced our understanding of the ENS and its interactions with the intestinal epithelium. Workman et al. demonstrated a groundbreaking approach by differentiating human ESC into ENS cells after four weeks of in vitro culture. These cells were then co-cultured with HIOs derived from human embryonic and iPSC. The process involved differentiating the stem cells into definitive endoderm, followed by mid/hindgut tube spheroids, and, finally, organoids [80]. When these ENS-containing HIOs were transplanted into mice, they exhibited a resemblance to the adult ganglia morphology observed in the myenteric and submucosal plexuses. Likewise, another study devised a method for constructing organoids by incorporating enteric neuroglial, mesenchymal, and epithelial precursors, all of which were derived from iPSCs and subsequently differentiated [222]. However, it is worth considering alternative models, such as murine or human models, that involve differentiating crypts to form organoids and isolating neurons from submucosal and myenteric plexuses, which offer certain advantages in terms of neuronal complexity. However, further characterization of the neuronal types and their functionality is needed to fully assess the potential of this technique. Furthermore, relying solely on organoids based on the crypt–villus unit does not fully represent the complexity of the neuronal interactions in the gut. Therefore, expanding the scope of the co-culture model to include components beyond the crypt–villus unit, including additional cell types such as smooth muscle cells or ICCs, into the co-culture system could improve the accuracy and representation of the physiological environment of the gut. To advance our understanding of the interaction between the GI tract and the ENS and achieve breakthroughs in this field, we recommend the use of our developed co-culture system. For further guidance, we recommend consulting the companion protocol article titled ‘Isolation of Myenteric and Submucosal Plexus from Mouse Gastrointestinal Tract and Subsequent Co-culture with Small Intestinal Organoids.’ This article describes a novel protocol for the isolation of myenteric and submucosal plexuses from the mouse GI tract and their subsequent co-culture with small intestinal organoids.

## 2. Conclusions

The imperative for developing co-culture systems, integrating myenteric and submucosal neurons with intestinal organoids, arises from the essential need to establish transformative models for GI diseases. These include neuropathies, liver diseases, and other extra-intestinal conditions influenced by the intestinal microbiome or immune system. The aim is to understand the physiopathological processes and pioneer innovative therapeutic interventions. These co-culture techniques promise to revolutionize the treatment landscape for GI diseases by providing accessible models, defining new molecular targets, and facilitating the development and testing of innovative therapies. Additionally, by integrating myenteric and submucosal neurons with organoids, these systems reveal benefits that extend beyond these aspects.

The integration of myenteric neurons, submucosal neurons, and organoids is crucial to open up avenues for exploring the communication between neurons and the gut microbiota. These endeavors hold the potential to provide valuable insights into the microbiome’s role in intestinal health and disease. Moreover, this type of integrated model is needed to study the influence of specific pathobionts. Another benefit of creating and improving these systems is reducing the reliance on germ-free mice and enhancing the cost-effectiveness of molecular mechanism investigations. It would also allow the generation of other complex co-culture systems by integrating isolated intestinal immune cells to understand microbiome–neuroimmune interactions. Additionally, expanding the scope of the co-culture model to integrate components beyond the crypt–villus unit, such as smooth muscle cells or ICCs, holds the potential for better replicating the microenvironment crucial for shaping the phenotype of the enteric neurons. These elements could enhance the accuracy and fidelity of the gut’s physiological representation.

The emerging field of ENS stem cell therapies for enteric neuropathies, along with the use of intestinal organoids in regenerative medicine for GI diseases, holds promise. The integration of co-cultured ENS–intestinal organoids with preestablished innervations could offer new alternatives in regenerative medicine. Leveraging patient-derived autologous cells may eliminate the need for immune suppression, presenting a revolutionary step forward. Refining cell isolation, culture methods, and engineering strategies is a must for substantial progress in the field.

As we delve into the complex interactions within the intestinal mucosal barrier, the creation of ENS models considering both myenteric and submucosal neurons and co-culturing them with intestinal organoids represents a groundbreaking advance in organoid technology. While successful clinical translation remains a future goal, improvements in multicellular co-culture systems, including material and technical enhancements, are essential for better simulating the in vivo intestinal environment and establishing efficacy. The development of this novel system is imperative, as it offers promise for future research and the treatment of digestive diseases, signifying a significant leap toward revolutionizing patient outcomes.

## Abbreviations

3D, three-dimensional; 5-HT, 5-hydroxytryptamine or serotonin; ACh, acetylcholine; ADPR, adenosine 5′-diphosphate ribose; AhR, aryl hydrocarbon receptor; Akt, protein kinase B; ALD, alcohol-associated liver disease; Ang4, angiopoietin-4; APC, antigen-presenting cells; ATP, adenosine triphosphate; BMP, bone morphogenetic protein; BNC2, zinc finger protein basonuclin-2; Cas 9, CRISPR-associated protein 9; CBCs, crypt base columnar cells; CCR6, C-C chemokine receptor type 6; CHAT, choline acetyltransferase; CRISPR-Cas9, Clustered Regularly Interspaced Short Palindromic Repeats-Cas9; CR, calretinin; CXCL8, C-X-C motif chemokine ligand 8; CXCR1, C-X-C chemokine receptor type 1; DAPI, 4′,6-diamidino-2-phenylindole; ECM, extracellular matrix; EEC, enteroendocrine cells; EGF, epidermal growth factor; ENS, enteric nervous system; Erk1/2, extracellular signal-regulated kinase 1 and 2; ESC, embryonic stem cells; FFA, free fatty acid receptor; GALT, gut-associated lymphoid tissue; GAPs, goblet cell-associated antigen passages; GI, gastrointestinal; HIO, human intestinal organoid; IBD, inflammatory bowel disease; IBS, irritable bowel syndrome; ICC, interstitial cells of Cajal; IEC, intestinal epithelial cells; IgA, immunoglobulin A; iHNO, induced human normal organoid model; iHUCO, induced human UC-derived organoid, IL-17, interleukin-17; IL-18, interleukin-18; IL-22, interleukin-22; ILC3, type 3 innate lymphoid cells, ILC3s, type 3 innate lymphoid cells; ILCs, innate lymphoid cells, IPANs, intrinsic primary afferent neurons; iPSC, induced pluripotent stem cell; ISC, intestinal stem cells; ISEMFs, intestinal subepithelial myofibroblasts; Itln1, intelecitin-1; LGR5+, leucine-rich repeat-containing G protein-coupled receptor 5; M cells, intestinal microfold cells, M2, muscarinic acetylcholine receptor 2; M4, muscarinic acetylcholine receptor 4; MAPKs, mitogen-activated protein kinase; MIRACL-seq, mining rare cells sequencing; MMTV, mammary tumor virus; NCCs, neural crest cells; NF-κB, nuclear factor kappa-light-chain-enhancer of activated B cells; nNOS, neuronal nitric oxide synthase; NO, nitric oxide; NOS, vesicular acetylcholine transporter; PACAP, pituitary adenylate cyclase-activating polypeptide; PACAP, pituitary adenylate cyclase-activating polypeptide; PBX3, PBX homeobox 3; PDGFRα, platelet-derived growth factor receptor α positive; PI3K, phosphoinositide 3-kinase; RBFOX1, RAISIN RNA-seq, ribosomes and intact single nucleus; RNA biding fox-1 homolog 1; Retnlb, resistin-like molecule beta; RORγ+, retinoic acid receptor-related orphan receptor gamma; SATB1, SATB homeobox 1; scRNAseq, single-cell RNA sequencing; SFB, segmented filamentous bacteria; SIP, smooth muscle cells/ICC/PDGFRα+ syncytium; snRNA-seq, single-nucleus RNA-seq; SOM, somatostatin; TA cells, transit-amplifying cells; TBX2, T-box transcription factor 2; TBX3, T-box transcription factor 3; TGFβ, transforming growth factor beta; TGR5, G-protein coupled bile acid receptor 5; TK, tachykinin; TLRs, toll-like receptors; TMR, tetramethylrhodamine-dextran; Treg cells, regulatory T cells; TUBB3, neuron marker β-tubulin III; UC, ulcerative colitis; UEA-I, Ulex Europaeus Agglutinin I; VIP, vasoactive intestinal peptide; VIPR2, VIP receptor type 2; Wnt3a, wingless-type MMTV integration site family member 3A;YFP, yellow fluorescent protein; α2β4 nAChR, alpha-2 beta-4 nicotinic acetylcholine receptor; β-NAD, β-nicotinamide adenine dinucleotide.

## References

[1] Bruellman R., Llorente C. (2021). A Perspective Of Intestinal Immune-Microbiome Interactions In Alcohol-Associated Liver Disease. Int. J. Biol. Sci..

[2] Sharkey K.A., Mawe G.M. (2023). The enteric nervous system. Physiol. Rev..

[3] Said H.M. (2012). Physiology of the Gastrointestinal Tract, Two Volume Set.

[4] Furness J.B. (2012). The enteric nervous system and neurogastroenterology. Nat. Rev. Gastroenterol. Hepatol..

[5] Spencer N.J., Hu H. (2020). Enteric nervous system: Sensory transduction, neural circuits and gastrointestinal motility. Nat. Rev. Gastroenterol. Hepatol..

[6] Hens J., Gajda M., Scheuermann D.W., Adriaensen D., Timmermans J.P. (2002). The longitudinal smooth muscle layer of the pig small intestine is innervated by both myenteric and submucous neurons. Histochem. Cell Biol..

[7] Porter A.J., Wattchow D.A., Brookes S.J., Costa M. (1999). Projections of nitric oxide synthase and vasoactive intestinal polypeptide-reactive submucosal neurons in the human colon. J. Gastroenterol. Hepatol..

[8] Wang H., Foong J.P.P., Harris N.L., Bornstein J.C. (2022). Enteric neuroimmune interactions coordinate intestinal responses in health and disease. Mucosal Immunol..

[9] Brehmer A. (2021). Classification of human enteric neurons. Histochem. Cell Biol..

[10] Foong J.P.P., Hung L.Y., Poon S., Savidge T.C., Bornstein J.C. (2020). Early life interaction between the microbiota and the enteric nervous system. Am. J. Physiol. Gastrointest. Liver Physiol..

[11] Furness J.B. (2008). The Enteric Nervous System.

[12] Maloy K.J., Powrie F. (2011). Intestinal homeostasis and its breakdown in inflammatory bowel disease. Nature.

[13] Schnabl B. (2013). Linking intestinal homeostasis and liver disease. Curr. Opin. Gastroenterol..

[14] Cani P.D. (2017). Gut microbiota—At the intersection of everything?. Nat. Rev. Gastroenterol. Hepatol..

[15] Silva M., Brunner V., Tschurtschenthaler M. (2021). Microbiota and Colorectal Cancer: From Gut to Bedside. Front. Pharmacol..

[16] Okumura R., Takeda K. (2017). Roles of intestinal epithelial cells in the maintenance of gut homeostasis. Exp. Mol. Med..

[17] Mowat A.M., Agace W.W. (2014). Regional specialization within the intestinal immune system. Nat. Rev. Immunol..

[18] Fang J., Wang H., Zhou Y., Zhang H., Zhou H., Zhang X. (2021). Slimy partners: The mucus barrier and gut microbiome in ulcerative colitis. Exp. Mol. Med..

[19] Vaishnava S., Yamamoto M., Severson K.M., Ruhn K.A., Yu X., Koren O., Ley R., Wakeland E.K., Hooper L.V. (2011). The antibacterial lectin RegIIIgamma promotes the spatial segregation of microbiota and host in the intestine. Science.

[20] Roos S., Jonsson H. (2002). A high-molecular-mass cell-surface protein from Lactobacillus reuteri 1063 adheres to mucus components. Microbiol..

[21] Pacheco A.R., Curtis M.M., Ritchie J.M., Munera D., Waldor M.K., Moreira C.G., Sperandio V. (2012). Fucose sensing regulates bacterial intestinal colonization. Nature.

[22] Furter M., Sellin M.E., Hansson G.C., Hardt W.D. (2019). Mucus Architecture and Near-Surface Swimming Affect Distinct Salmonella Typhimurium Infection Patterns along the Murine Intestinal Tract. Cell Rep..

[23] Huang Y.L., Chassard C., Hausmann M., von Itzstein M., Hennet T. (2015). Sialic acid catabolism drives intestinal inflammation and microbial dysbiosis in mice. Nat. Commun..

[24] Kini A., Singh A.K., Riederer B., Yang I., Tan X., di Stefano G., Tan Q., Xiao F., Xia W., Suerbaum S. (2020). Slc26a3 deletion alters pH-microclimate, mucin biosynthesis, microbiome composition and increases the TNFalpha expression in murine colon. Acta Physiol..

[25] Swidsinski A., Sydora B.C., Doerffel Y., Loening-Baucke V., Vaneechoutte M., Lupicki M., Scholze J., Lochs H., Dieleman L.A. (2007). Viscosity gradient within the mucus layer determines the mucosal barrier function and the spatial organization of the intestinal microbiota. Inflamm. Bowel Dis..

[26] Hooper L.V., Stappenbeck T.S., Hong C.V., Gordon J.I. (2003). Angiogenins: A new class of microbicidal proteins involved in innate immunity. Nat. Immunol..

[27] Barr J.J., Auro R., Furlan M., Whiteson K.L., Erb M.L., Pogliano J., Stotland A., Wolkowicz R., Cutting A.S., Doran K.S. (2013). Bacteriophage adhering to mucus provide a non-host-derived immunity. Proc. Natl. Acad. Sci. USA.

[28] Thoma C. (2018). Bacteriophage virome in IBD. Nat. Rev. Gastroenterol. Hepatol..

[29] Liang S.C., Tan X.Y., Luxenberg D.P., Karim R., Dunussi-Joannopoulos K., Collins M., Fouser L.A. (2006). Interleukin (IL)-22 and IL-17 are coexpressed by Th17 cells and cooperatively enhance expression of antimicrobial peptides. J. Exp. Med..

[30] Vonarbourg C., Mortha A., Bui V.L., Hernandez P.P., Kiss E.A., Hoyler T., Flach M., Bengsch B., Thimme R., Holscher C. (2010). Regulated expression of nuclear receptor RORgammat confers distinct functional fates to NK cell receptor-expressing RORgammat(+) innate lymphocytes. Immunity.

[31] Kim S.V., Xiang W.V., Kwak C., Yang Y., Lin X.W., Ota M., Sarpel U., Rifkin D.B., Xu R., Littman D.R. (2013). GPR15-mediated homing controls immune homeostasis in the large intestine mucosa. Science.

[32] Smith P.M., Howitt M.R., Panikov N., Michaud M., Gallini C.A., Bohlooly Y.M., Glickman J.N., Garrett W.S. (2013). The microbial metabolites, short-chain fatty acids, regulate colonic Treg cell homeostasis. Science.

[33] Hoytema van Konijnenburg D.P., Reis B.S., Pedicord V.A., Farache J., Victora G.D., Mucida D. (2017). Intestinal Epithelial and Intraepithelial T Cell Crosstalk Mediates a Dynamic Response to Infection. Cell.

[34] Seguella L., McClain J.L., Esposito G., Gulbransen B.D. (2022). Functional Intraregional and Interregional Heterogeneity between Myenteric Glial Cells of the Colon and Duodenum in Mice. J. Neurosci..

[35] Wang R., Li Z., Liu S., Zhang D. (2023). Global, regional, and national burden of 10 digestive diseases in 204 countries and territories from 1990 to 2019. Front. Public Health.

[36] Sun H., Chow E.C., Liu S., Du Y., Pang K.S. (2008). The Caco-2 cell monolayer: Usefulness and limitations. Expert. Opin. Drug Metab. Toxicol..

[37] Rousset M. (1986). The human colon carcinoma cell lines HT-29 and Caco-2: Two in vitro models for the study of intestinal differentiation. Biochimie.

[38] Murakami H., Masui H. (1980). Hormonal control of human colon carcinoma cell growth in serum-free medium. Proc. Natl. Acad. Sci. USA.

[39] Sato T., Vries R.G., Snippert H.J., van de Wetering M., Barker N., Stange D.E., van Es J.H., Abo A., Kujala P., Peters P.J. (2009). Single Lgr5 stem cells build crypt-villus structures in vitro without a mesenchymal niche. Nature.

[40] Clevers H. (2016). Modeling Development and Disease with Organoids. Cell.

[41] Spence J.R., Mayhew C.N., Rankin S.A., Kuhar M.F., Vallance J.E., Tolle K., Hoskins E.E., Kalinichenko V.V., Wells S.I., Zorn A.M. (2011). Directed differentiation of human pluripotent stem cells into intestinal tissue in vitro. Nature.

[42] Thomson J.A., Itskovitz-Eldor J., Shapiro S.S., Waknitz M.A., Swiergiel J.J., Marshall V.S., Jones J.M. (1998). Embryonic stem cell lines derived from human blastocysts. Science.

[43] Stelzner M., Helmrath M., Dunn J.C., Henning S.J., Houchen C.W., Kuo C., Lynch J., Li L., Magness S.T., Martin M.G. (2012). A nomenclature for intestinal in vitro cultures. Am. J. Physiol. Gastrointest. Liver Physiol..

[44] Cheng H., Leblond C.P. (1974). Origin, differentiation and renewal of the four main epithelial cell types in the mouse small intestine. V. Unitarian Theory of the origin of the four epithelial cell types. Am. J. Anat..

[45] Sato T., Stange D.E., Ferrante M., Vries R.G., Van Es J.H., Van den Brink S., Van Houdt W.J., Pronk A., Van Gorp J., Siersema P.D. (2011). Long-term expansion of epithelial organoids from human colon, adenoma, adenocarcinoma, and Barrett’s epithelium. Gastroenterology.

[46] Sato T., Clevers H. (2013). Growing self-organizing mini-guts from a single intestinal stem cell: Mechanism and applications. Science.

[47] Sato T., van Es J.H., Snippert H.J., Stange D.E., Vries R.G., van den Born M., Barker N., Shroyer N.F., van de Wetering M., Clevers H. (2011). Paneth cells constitute the niche for Lgr5 stem cells in intestinal crypts. Nature.

[48] Miyoshi H., Stappenbeck T.S. (2013). In vitro expansion and genetic modification of gastrointestinal stem cells in spheroid culture. Nat. Protoc..

[49] McCracken K.W., Howell J.C., Wells J.M., Spence J.R. (2011). Generating human intestinal tissue from pluripotent stem cells in vitro. Nat. Protoc..

[50] Hofer M., Lutolf M.P. (2021). Engineering organoids. Nat. Rev. Mater..

[51] Fu J., Warmflash A., Lutolf M.P. (2021). Stem-cell-based embryo models for fundamental research and translation. Nat. Mater..

[52] Brassard J.A., Lutolf M.P. (2019). Engineering Stem Cell Self-organization to Build Better Organoids. Cell Stem Cell.

[53] Kasendra M., Tovaglieri A., Sontheimer-Phelps A., Jalili-Firoozinezhad S., Bein A., Chalkiadaki A., Scholl W., Zhang C., Rickner H., Richmond C.A. (2018). Development of a primary human Small Intestine-on-a-Chip using biopsy-derived organoids. Sci. Rep..

[54] Jalili-Firoozinezhad S., Gazzaniga F.S., Calamari E.L., Camacho D.M., Fadel C.W., Bein A., Swenor B., Nestor B., Cronce M.J., Tovaglieri A. (2019). A complex human gut microbiome cultured in an anaerobic intestine-on-a-chip. Nat. Biomed. Eng..

[55] Michiba K., Maeda K., Shimomura O., Miyazaki Y., Hashimoto S., Oda T., Kusuhara H. (2022). Usefulness of Human Jejunal Spheroid-Derived Differentiated Intestinal Epithelial Cells for the Prediction of Intestinal Drug Absorption in Humans. Drug Metab. Dispos..

[56] Dedhia P.H., Bertaux-Skeirik N., Zavros Y., Spence J.R. (2016). Organoid Models of Human Gastrointestinal Development and Disease. Gastroenterology.

[57] Bartfeld S., Clevers H. (2015). Organoids as Model for Infectious Diseases: Culture of Human and Murine Stomach Organoids and Microinjection of Helicobacter Pylori. J. Vis. Exp..

[58] Almeqdadi M., Mana M.D., Roper J., Yilmaz O.H. (2019). Gut organoids: Mini-tissues in culture to study intestinal physiology and disease. Am. J. Physiol. Cell Physiol..

[59] Dekkers J.F., Wiegerinck C.L., de Jonge H.R., Bronsveld I., Janssens H.M., de Winter-de Groot K.M., Brandsma A.M., de Jong N.W., Bijvelds M.J., Scholte B.J. (2013). A functional CFTR assay using primary cystic fibrosis intestinal organoids. Nat. Med..

[60] Schwank G., Koo B.K., Sasselli V., Dekkers J.F., Heo I., Demircan T., Sasaki N., Boymans S., Cuppen E., van der Ent C.K. (2013). Functional repair of CFTR by CRISPR/Cas9 in intestinal stem cell organoids of cystic fibrosis patients. Cell Stem Cell.

[61] Drost J., van Jaarsveld R.H., Ponsioen B., Zimberlin C., van Boxtel R., Buijs A., Sachs N., Overmeer R.M., Offerhaus G.J., Begthel H. (2015). Sequential cancer mutations in cultured human intestinal stem cells. Nature.

[62] Matano M., Date S., Shimokawa M., Takano A., Fujii M., Ohta Y., Watanabe T., Kanai T., Sato T. (2015). Modeling colorectal cancer using CRISPR-Cas9-mediated engineering of human intestinal organoids. Nat. Med..

[63] Giandomenico S.L., Mierau S.B., Gibbons G.M., Wenger L.M.D., Masullo L., Sit T., Sutcliffe M., Boulanger J., Tripodi M., Derivery E. (2019). Cerebral organoids at the air-liquid interface generate diverse nerve tracts with functional output. Nat. Neurosci..

[64] Jaganathan H., Gage J., Leonard F., Srinivasan S., Souza G.R., Dave B., Godin B. (2014). Three-dimensional in vitro co-culture model of breast tumor using magnetic levitation. Sci. Rep..

[65] Garreta E., Kamm R.D., Chuva de Sousa Lopes S.M., Lancaster M.A., Weiss R., Trepat X., Hyun I., Montserrat N. (2021). Rethinking organoid technology through bioengineering. Nat. Mater..

[66] Asano Y., Nishiguchi A., Matsusaki M., Okano D., Saito E., Akashi M., Shimoda H. (2014). Ultrastructure of blood and lymphatic vascular networks in three-dimensional cultured tissues fabricated by extracellular matrix nanofilm-based cell accumulation technique. Microscopy.

[67] Sugimoto S., Sato T. (2017). Establishment of 3D Intestinal Organoid Cultures from Intestinal Stem Cells. Methods Mol. Biol..

[68] Huang L., Xiao L., Jung Poudel A., Li J., Zhou P., Gauthier M., Liu H., Wu Z., Yang G. (2018). Porous chitosan microspheres as microcarriers for 3D cell culture. Carbohydr. Polym..

[69] Yui S., Nakamura T., Sato T., Nemoto Y., Mizutani T., Zheng X., Ichinose S., Nagaishi T., Okamoto R., Tsuchiya K. (2012). Functional engraftment of colon epithelium expanded in vitro from a single adult Lgr5(+) stem cell. Nat. Med..

[70] Yui S., Azzolin L., Maimets M., Pedersen M.T., Fordham R.P., Hansen S.L., Larsen H.L., Guiu J., Alves M.R.P., Rundsten C.F. (2018). YAP/TAZ-Dependent Reprogramming of Colonic Epithelium Links ECM Remodeling to Tissue Regeneration. Cell Stem Cell.

[71] Fukuda M., Mizutani T., Mochizuki W., Matsumoto T., Nozaki K., Sakamaki Y., Ichinose S., Okada Y., Tanaka T., Watanabe M. (2014). Small intestinal stem cell identity is maintained with functional Paneth cells in heterotopically grafted epithelium onto the colon. Genes Dev..

[72] Sugimoto S., Ohta Y., Fujii M., Matano M., Shimokawa M., Nanki K., Date S., Nishikori S., Nakazato Y., Nakamura T. (2018). Reconstruction of the Human Colon Epithelium In Vivo. Cell Stem Cell.

[73] Zhu Y., Yang S., Zhao N., Liu C., Zhang F., Guo Y., Liu H. (2021). CXCL8 chemokine in ulcerative colitis. Biomed. Pharmacother..

[74] Sarvestani S.K., Signs S., Hu B., Yeu Y., Feng H., Ni Y., Hill D.R., Fisher R.C., Ferrandon S., DeHaan R.K. (2021). Induced organoids derived from patients with ulcerative colitis recapitulate colitic reactivity. Nat. Commun..

[75] Gunther C., Winner B., Neurath M.F., Stappenbeck T.S. (2022). Organoids in gastrointestinal diseases: From experimental models to clinical translation. Gut.

[76] Wilke G., Funkhouser-Jones L.J., Wang Y., Ravindran S., Wang Q., Beatty W.L., Baldridge M.T., VanDussen K.L., Shen B., Kuhlenschmidt M.S. (2019). A Stem-Cell-Derived Platform Enables Complete Cryptosporidium Development In Vitro and Genetic Tractability. Cell Host Microbe.

[77] Puschhof J., Pleguezuelos-Manzano C., Martinez-Silgado A., Akkerman N., Saftien A., Boot C., de Waal A., Beumer J., Dutta D., Heo I. (2021). Intestinal organoid cocultures with microbes. Nat. Protoc..

[78] Bar-Ephraim Y.E., Kretzschmar K., Clevers H. (2020). Organoids in immunological research. Nat. Rev. Immunol..

[79] Schreurs R., Baumdick M.E., Drewniak A., Bunders M.J. (2021). In vitro co-culture of human intestinal organoids and lamina propria-derived CD4(+) T cells. STAR Protoc..

[80] Workman M.J., Mahe M.M., Trisno S., Poling H.M., Watson C.L., Sundaram N., Chang C.F., Schiesser J., Aubert P., Stanley E.G. (2017). Engineered human pluripotent-stem-cell-derived intestinal tissues with a functional enteric nervous system. Nat. Med..

[81] McCauley H.A., Wells J.M. (2017). Pluripotent stem cell-derived organoids: Using principles of developmental biology to grow human tissues in a dish. Development.

[82] Lahar N., Lei N.Y., Wang J., Jabaji Z., Tung S.C., Joshi V., Lewis M., Stelzner M., Martin M.G., Dunn J.C. (2011). Intestinal subepithelial myofibroblasts support in vitro and in vivo growth of human small intestinal epithelium. PLoS ONE.

[83] Fattahi F., Steinbeck J.A., Kriks S., Tchieu J., Zimmer B., Kishinevsky S., Zeltner N., Mica Y., El-Nachef W., Zhao H. (2016). Deriving human ENS lineages for cell therapy and drug discovery in Hirschsprung disease. Nature.

[84] Noel G., Baetz N.W., Staab J.F., Donowitz M., Kovbasnjuk O., Pasetti M.F., Zachos N.C. (2017). A primary human macrophage-enteroid co-culture model to investigate mucosal gut physiology and host-pathogen interactions. Sci. Rep..

[85] Shaffiey S.A., Jia H., Keane T., Costello C., Wasserman D., Quidgley M., Dziki J., Badylak S., Sodhi C.P., March J.C. (2016). Intestinal stem cell growth and differentiation on a tubular scaffold with evaluation in small and large animals. Regen. Med..

[86] In J., Foulke-Abel J., Zachos N.C., Hansen A.M., Kaper J.B., Bernstein H.D., Halushka M., Blutt S., Estes M.K., Donowitz M. (2016). Enterohemorrhagic Escherichia coli reduce mucus and intermicrovillar bridges in human stem cell-derived colonoids. Cell Mol. Gastroenterol. Hepatol..

[87] Zhang Y.G., Wu S., Xia Y., Sun J. (2014). Salmonella-infected crypt-derived intestinal organoid culture system for host-bacterial interactions. Physiol. Rep..

[88] Leslie J.L., Huang S., Opp J.S., Nagy M.S., Kobayashi M., Young V.B., Spence J.R. (2015). Persistence and toxin production by Clostridium difficile within human intestinal organoids result in disruption of epithelial paracellular barrier function. Infect. Immun..

[89] Heo I., Dutta D., Schaefer D.A., Iakobachvili N., Artegiani B., Sachs N., Boonekamp K.E., Bowden G., Hendrickx A.P.A., Willems R.J.L. (2018). Modelling Cryptosporidium infection in human small intestinal and lung organoids. Nat. Microbiol..

[90] Finkbeiner S.R., Zeng X.L., Utama B., Atmar R.L., Shroyer N.F., Estes M.K. (2012). Stem cell-derived human intestinal organoids as an infection model for rotaviruses. mBio.

[91] Saxena K., Blutt S.E., Ettayebi K., Zeng X.L., Broughman J.R., Crawford S.E., Karandikar U.C., Sastri N.P., Conner M.E., Opekun A.R. (2016). Human Intestinal Enteroids: A New Model To Study Human Rotavirus Infection, Host Restriction, and Pathophysiology. J. Virol..

[92] Ettayebi K., Crawford S.E., Murakami K., Broughman J.R., Karandikar U., Tenge V.R., Neill F.H., Blutt S.E., Zeng X.L., Qu L. (2016). Replication of human noroviruses in stem cell-derived human enteroids. Science.

[93] Holloway E.M., Capeling M.M., Spence J.R. (2019). Biologically inspired approaches to enhance human organoid complexity. Development.

[94] Kim S., Cho A.N., Min S., Kim S., Cho S.W. (2019). Organoids for Advanced Therapeutics and Disease Models. Adv. Ther..

[95] Sarker M.D., Naghieh S., Sharma N.K., Chen X. (2018). 3D biofabrication of vascular networks for tissue regeneration: A report on recent advances. J. Pharm. Anal..

[96] Zhuang P., Sun A.X., An J., Chua C.K., Chew S.Y. (2018). 3D neural tissue models: From spheroids to bioprinting. Biomaterials.

[97] Yamaoka N., Shimizu K., Imaizumi Y., Ito T., Okada Y., Honda H. (2019). Open-Chamber Co-Culture Microdevices for Single-Cell Analysis of Skeletal Muscle Myotubes and Motor Neurons with Neuromuscular Junctions. Biochip J..

[98] Kim E., Choi S., Kang B., Kong J., Kim Y., Yoon W.H., Lee H.R., Kim S., Kim H.M., Lee H. (2020). Creation of bladder assembloids mimicking tissue regeneration and cancer. Nature.

[99] Nashimoto Y., Hayashi T., Kunita I., Nakamasu A., Torisawa Y., Nakayama M., Takigawa-Imamura H., Kotera H., Nishiyama K., Miura T. (2017). Integrating perfusable vascular networks with a three-dimensional tissue in a microfluidic device. Integr. Biol..

[100] Park S.E., Georgescu A., Huh D. (2019). Organoids-on-a-chip. Science.

[101] Shin W., Kim H.J. (2022). 3D in vitro morphogenesis of human intestinal epithelium in a gut-on-a-chip or a hybrid chip with a cell culture insert. Nat. Protoc..

[102] Liu H., Wang Y., Cui K., Guo Y., Zhang X., Qin J. (2019). Advances in Hydrogels in Organoids and Organs-on-a-Chip. Adv. Mater..

[103] Kratochvil M.J., Seymour A.J., Li T.L., Pasca S.P., Kuo C.J., Heilshorn S.C. (2019). Engineered materials for organoid systems. Nat. Rev. Mater..

[104] Co J.Y., Margalef-Catala M., Monack D.M., Amieva M.R. (2021). Controlling the polarity of human gastrointestinal organoids to investigate epithelial biology and infectious diseases. Nat. Protoc..

[105] Stroulios G., Stahl M., Elstone F., Chang W., Louis S., Eaves A., Simmini S., Conder R.K. (2021). Culture Methods to Study Apical-Specific Interactions using Intestinal Organoid Models. J. Vis. Exp..

[106] Zhang J., Hernandez-Gordillo V., Trapecar M., Wright C., Taketani M., Schneider K., Chen W.L.K., Stas E., Breault D.T., Carrier R.L. (2021). Coculture of primary human colon monolayer with human gut bacteria. Nat. Protoc..

[107] Sayed I.M., Tindle C., Fonseca A.G., Ghosh P., Das S. (2021). Functional assays with human patient-derived enteroid monolayers to assess the human gut barrier. STAR Protoc..

[108] Patel K.K., Miyoshi H., Beatty W.L., Head R.D., Malvin N.P., Cadwell K., Guan J.L., Saitoh T., Akira S., Seglen P.O. (2013). Autophagy proteins control goblet cell function by potentiating reactive oxygen species production. EMBO J..

[109] Zeve D., Stas E., de Sousa Casal J., Mannam P., Qi W., Yin X., Dubois S., Shah M.S., Syverson E.P., Hafner S. (2022). Robust differentiation of human enteroendocrine cells from intestinal stem cells. Nat. Commun..

[110] Miyoshi H., VanDussen K.L., Malvin N.P., Ryu S.H., Wang Y., Sonnek N.M., Lai C.W., Stappenbeck T.S. (2017). Prostaglandin E2 promotes intestinal repair through an adaptive cellular response of the epithelium. EMBO J..

[111] Kanaya T., Sakakibara S., Jinnohara T., Hachisuka M., Tachibana N., Hidano S., Kobayashi T., Kimura S., Iwanaga T., Nakagawa T. (2018). Development of intestinal M cells and follicle-associated epithelium is regulated by TRAF6-mediated NF-kappaB signaling. J. Exp. Med..

[112] Costa M., Brookes S.J., Hennig G.W. (2000). Anatomy and physiology of the enteric nervous system. Gut.

[113] Reigstad C.S., Salmonson C.E., Rainey J.F., Szurszewski J.H., Linden D.R., Sonnenburg J.L., Farrugia G., Kashyap P.C. (2015). Gut microbes promote colonic serotonin production through an effect of short-chain fatty acids on enterochromaffin cells. FASEB J..

[114] Walther D.J., Peter J.U., Bashammakh S., Hortnagl H., Voits M., Fink H., Bader M. (2003). Synthesis of serotonin by a second tryptophan hydroxylase isoform. Science.

[115] Lyte M. (2011). Probiotics function mechanistically as delivery vehicles for neuroactive compounds: Microbial endocrinology in the design and use of probiotics. Bioessays.

[116] Parathan P., Wang Y., Leembruggen A.J., Bornstein J.C., Foong J.P. (2020). The enteric nervous system undergoes significant chemical and synaptic maturation during adolescence in mice. Dev. Biol..

[117] Avetisyan M., Wang H., Schill E.M., Bery S., Grider J.R., Hassell J.A., Stappenbeck T., Heuckeroth R.O. (2015). Hepatocyte Growth Factor and MET Support Mouse Enteric Nervous System Development, the Peristaltic Response, and Intestinal Epithelial Proliferation in Response to Injury. J. Neurosci..

[118] Erickson C.S., Lee S.J., Barlow-Anacker A.J., Druckenbrod N.R., Epstein M.L., Gosain A. (2014). Appearance of cholinergic myenteric neurons during enteric nervous system development: Comparison of different ChAT fluorescent mouse reporter lines. Neurogastroenterol. Motil..

[119] Qu Z.D., Thacker M., Castelucci P., Bagyanszki M., Epstein M.L., Furness J.B. (2008). Immunohistochemical analysis of neuron types in the mouse small intestine. Cell Tissue Res..

[120] Foong J.P., Tough I.R., Cox H.M., Bornstein J.C. (2014). Properties of cholinergic and non-cholinergic submucosal neurons along the mouse colon. J. Physiol..

[121] Morarach K., Mikhailova A., Knoflach V., Memic F., Kumar R., Li W., Ernfors P., Marklund U. (2021). Diversification of molecularly defined myenteric neuron classes revealed by single-cell RNA sequencing. Nat. Neurosci..

[122] Drokhlyansky E., Smillie C.S., Van Wittenberghe N., Ericsson M., Griffin G.K., Eraslan G., Dionne D., Cuoco M.S., Goder-Reiser M.N., Sharova T. (2020). The Human and Mouse Enteric Nervous System at Single-Cell Resolution. Cell.

[123] Wright C.M., Schneider S., Smith-Edwards K.M., Mafra F., Leembruggen A.J.L., Gonzalez M.V., Kothakapa D.R., Anderson J.B., Maguire B.A., Gao T. (2021). scRNA-Seq Reveals New Enteric Nervous System Roles for GDNF, NRTN, and TBX3. Cell Mol. Gastroenterol. Hepatol..

[124] May-Zhang A.A., Tycksen E., Southard-Smith A.N., Deal K.K., Benthal J.T., Buehler D.P., Adam M., Simmons A.J., Monaghan J.R., Matlock B.K. (2021). Combinatorial Transcriptional Profiling of Mouse and Human Enteric Neurons Identifies Shared and Disparate Subtypes In Situ. Gastroenterology.

[125] Lomax A.E., Furness J.B. (2000). Neurochemical classification of enteric neurons in the guinea-pig distal colon. Cell Tissue Res..

[126] Ochoa-Cortes F., Turco F., Linan-Rico A., Soghomonyan S., Whitaker E., Wehner S., Cuomo R., Christofi F.L. (2016). Enteric Glial Cells: A New Frontier in Neurogastroenterology and Clinical Target for Inflammatory Bowel Diseases. Inflamm. Bowel Dis..

[127] Kunze W.A., Bornstein J.C., Furness J.B. (1995). Identification of sensory nerve cells in a peripheral organ (the intestine) of a mammal. Neuroscience.

[128] Fung C., Vanden Berghe P. (2020). Functional circuits and signal processing in the enteric nervous system. Cell Mol. Life Sci..

[129] Furness J.B., Jones C., Nurgali K., Clerc N. (2004). Intrinsic primary afferent neurons and nerve circuits within the intestine. Prog. Neurobiol..

[130] Mazzuoli G., Schemann M. (2012). Mechanosensitive enteric neurons in the myenteric plexus of the mouse intestine. PLoS ONE.

[131] Neal K.B., Bornstein J.C. (2008). Targets of myenteric interneurons in the guinea-pig small intestine. Neurogastroenterol. Motil..

[132] Spencer N.J., Travis L., Wiklendt L., Costa M., Hibberd T.J., Brookes S.J., Dinning P., Hu H., Wattchow D.A., Sorensen J. (2021). Long range synchronization within the enteric nervous system underlies propulsion along the large intestine in mice. Commun. Biol..

[133] Furness J.B. (2000). Types of neurons in the enteric nervous system. J. Auton. Nerv. Syst..

[134] Costa M., Spencer N.J., Brookes S.J.H. (2021). The role of enteric inhibitory neurons in intestinal motility. Auton. Neurosci..

[135] Dickson E.J., Heredia D.J., Smith T.K. (2010). Critical role of 5-HT1A, 5-HT3, and 5-HT7 receptor subtypes in the initiation, generation, and propagation of the murine colonic migrating motor complex. Am. J. Physiol. Gastrointest. Liver Physiol..

[136] Burnstock G., Campbell G., Satchell D., Smythe A. (1970). Evidence that adenosine triphosphate or a related nucleotide is the transmitter substance released by non-adrenergic inhibitory nerves in the gut. Br. J. Pharmacol..

[137] Crist J.R., He X.D., Goyal R.K. (1992). Both ATP and the peptide VIP are inhibitory neurotransmitters in guinea-pig ileum circular muscle. J. Physiol..

[138] Xue L., Farrugia G., Sarr M.G., Szurszewski J.H. (1999). ATP is a mediator of the fast inhibitory junction potential in human jejunal circular smooth muscle. Am. J. Physiol..

[139] Sanders K.M., Koh S.D., Ro S., Ward S.M. (2012). Regulation of gastrointestinal motility—Insights from smooth muscle biology. Nat. Rev. Gastroenterol. Hepatol..

[140] Mutafova-Yambolieva V.N., Hwang S.J., Hao X., Chen H., Zhu M.X., Wood J.D., Ward S.M., Sanders K.M. (2007). Beta-nicotinamide adenine dinucleotide is an inhibitory neurotransmitter in visceral smooth muscle. Proc. Natl. Acad. Sci. USA.

[141] Durnin L., Hwang S.J., Ward S.M., Sanders K.M., Mutafova-Yambolieva V.N. (2012). Adenosine 5-diphosphate-ribose is a neural regulator in primate and murine large intestine along with beta-NAD(+). J. Physiol..

[142] Durnin L., Sanders K.M., Mutafova-Yambolieva V.N. (2013). Differential release of beta-NAD(+) and ATP upon activation of enteric motor neurons in primate and murine colons. Neurogastroenterol. Motil..

[143] Fujita A., Takeuchi T., Jun H., Hata F. (2003). Localization of Ca2+-activated K+ channel, SK3, in fibroblast-like cells forming gap junctions with smooth muscle cells in the mouse small intestine. J. Pharmacol. Sci..

[144] Komuro T., Seki K., Horiguchi K. (1999). Ultrastructural characterization of the interstitial cells of Cajal. Arch. Histol. Cytol..

[145] Torihashi S., Ward S.M., Nishikawa S., Nishi K., Kobayashi S., Sanders K.M. (1995). c-kit-dependent development of interstitial cells and electrical activity in the murine gastrointestinal tract. Cell Tissue Res..

[146] Ward S.M., Morris G., Reese L., Wang X.Y., Sanders K.M. (1998). Interstitial cells of Cajal mediate enteric inhibitory neurotransmission in the lower esophageal and pyloric sphincters. Gastroenterology.

[147] Kurahashi M., Zheng H., Dwyer L., Ward S.M., Koh S.D., Sanders K.M. (2011). A functional role for the ‘fibroblast-like cells’ in gastrointestinal smooth muscles. J. Physiol..

[148] Baker S.A., Hennig G.W., Salter A.K., Kurahashi M., Ward S.M., Sanders K.M. (2013). Distribution and Ca(^2+^) signalling of fibroblast-like (PDGFR(^+^)) cells in the murine gastric fundus. J. Physiol..

[149] Bornstein J.C., Costa M., Grider J.R. (2004). Enteric motor and interneuronal circuits controlling motility. Neurogastroenterol. Motil..

[150] Sia T.C., Flack N., Robinson L., Kyloh M., Nicholas S.J., Brookes S.J., Wattchow D.A., Dinning P., Oliver J., Spencer N.J. (2013). Is serotonin in enteric nerves required for distension-evoked peristalsis and propulsion of content in guinea-pig distal colon?. Neuroscience.

[151] Costa M., Wiklendt L., Simpson P., Spencer N.J., Brookes S.J., Dinning P.G. (2015). Neuromechanical factors involved in the formation and propulsion of fecal pellets in the guinea-pig colon. Neurogastroenterol. Motil..

[152] Rao M., Rastelli D., Dong L., Chiu S., Setlik W., Gershon M.D., Corfas G. (2017). Enteric Glia Regulate Gastrointestinal Motility but Are Not Required for Maintenance of the Epithelium in Mice. Gastroenterology.

[153] Obata Y., Castano A., Boeing S., Bon-Frauches A.C., Fung C., Fallesen T., de Aguero M.G., Yilmaz B., Lopes R., Huseynova A. (2020). Neuronal programming by microbiota regulates intestinal physiology. Nature.

[154] Muller P.A., Koscso B., Rajani G.M., Stevanovic K., Berres M.L., Hashimoto D., Mortha A., Leboeuf M., Li X.M., Mucida D. (2014). Crosstalk between muscularis macrophages and enteric neurons regulates gastrointestinal motility. Cell.

[155] Camilleri M. (2021). Gastrointestinal motility disorders in neurologic disease. J. Clin. Investig..

[156] Schonkeren S.L., Kuthe T.T., Idris M., Bon-Frauches A.C., Boesmans W., Melotte V. (2022). The gut brain in a dish: Murine primary enteric nervous system cell cultures. Neurogastroenterol. Motil..

[157] Hirst G.D., McKirdy H.C. (1975). Synaptic potentials recorded from neurones of the submucous plexus of guinea-pig small intestine. J. Physiol..

[158] Zhang Y., Hu W. (2013). Mouse enteric neuronal cell culture. Methods Mol. Biol..

[159] Accili E.A., Buchan A.M. (1996). Primary cell culture of human enteric neurons: Submucosal plexus. Methods Mol. Med..

[160] Hecking I., Stegemann L.N., Stahlke S., Theis V., Vorgerd M., Matschke V., Theiss C. (2023). Methods to Study the Myenteric Plexus of Rat Small Intestine. Cell Mol. Neurobiol..

[161] Barber K., Studer L., Fattahi F. (2019). Derivation of enteric neuron lineages from human pluripotent stem cells. Nat. Protoc..

[162] Puzan M., Hosic S., Ghio C., Koppes A. (2018). Enteric Nervous System Regulation of Intestinal Stem Cell Differentiation and Epithelial Monolayer Function. Sci. Rep..

[163] Anitha M., Joseph I., Ding X., Torre E.R., Sawchuk M.A., Mwangi S., Hochman S., Sitaraman S.V., Anania F., Srinivasan S. (2008). Characterization of fetal and postnatal enteric neuronal cell lines with improvement in intestinal neural function. Gastroenterology.

[164] Jessen K.R., McConnell J.D., Purves R.D., Burnstock G., Chamley-Campbell J. (1978). Tissue culture of mammalian enteric neurons. Brain Res..

[165] Bondurand N., Natarajan D., Thapar N., Atkins C., Pachnis V. (2003). Neuron and glia generating progenitors of the mammalian enteric nervous system isolated from foetal and postnatal gut cultures. Development.

[166] Schafer K.H., Hagl C.I., Rauch U. (2003). Differentiation of neurospheres from the enteric nervous system. Pediatr. Surg. Int..

[167] Nishi R., Willard A.L. (1985). Neurons dissociated from rat myenteric plexus retain differentiated properties when grown in cell culture. I. Morphological properties and immunocytochemical localization of transmitter candidates. Neuroscience.

[168] Korman L.Y., Nylen E.S., Finan T.M., Linnoila R.I., Becker K.L. (1988). Primary culture of the enteric nervous system from neonatal hamster intestine. Selection of vasoactive intestinal polypeptide-containing neurons. Gastroenterology.

[169] Burns A.J., Goldstein A.M., Newgreen D.F., Stamp L., Schafer K.H., Metzger M., Hotta R., Young H.M., Andrews P.W., Thapar N. (2016). White paper on guidelines concerning enteric nervous system stem cell therapy for enteric neuropathies. Dev. Biol..

[170] Brun P., Akbarali H.I. (2018). Culture of Neurons and Smooth Muscle Cells from the Myenteric Plexus of Adult Mice. Methods Mol. Biol..

[171] Smith T.H., Ngwainmbi J., Grider J.R., Dewey W.L., Akbarali H.I. (2013). An in-vitro preparation of isolated enteric neurons and glia from the myenteric plexus of the adult mouse. J. Vis. Exp..

[172] Wang Z., Ocadiz-Ruiz R., Sundaresan S., Ding L., Hayes M., Sahoo N., Xu H., Merchant J.L. (2018). Isolation of Enteric Glial Cells from the Submucosa and Lamina Propria of the Adult Mouse. J. Vis. Exp..

[173] Grundmann D., Klotz M., Rabe H., Glanemann M., Schafer K.H. (2015). Isolation of high-purity myenteric plexus from adult human and mouse gastrointestinal tract. Sci. Rep..

[174] Zhang Y., Hu W. (2021). Mouse Enteric Neuronal Cell Culture. Methods Mol. Biol..

[175] Wahba G., Hebert A.E., Grynspan D., Staines W., Schock S. (2016). A rapid and efficient method for dissociated cultures of mouse myenteric neurons. J. Neurosci. Methods.

[176] Ahrends T., Weiner M., Mucida D. (2022). Isolation of myenteric and submucosal plexus from mouse gastrointestinal tract and subsequent flow cytometry and immunofluorescence. STAR Protoc..

[177] Obata Y., Pachnis V. (2016). The Effect of Microbiota and the Immune System on the Development and Organization of the Enteric Nervous System. Gastroenterology.

[178] Yoo B.B., Mazmanian S.K. (2017). The Enteric Network: Interactions between the Immune and Nervous Systems of the Gut. Immunity.

[179] Bubeck M., Becker C., Patankar J.V. (2023). Guardians of the gut: Influence of the enteric nervous system on the intestinal epithelial barrier. Front. Med..

[180] Westphalen C.B., Asfaha S., Hayakawa Y., Takemoto Y., Lukin D.J., Nuber A.H., Brandtner A., Setlik W., Remotti H., Muley A. (2014). Long-lived intestinal tuft cells serve as colon cancer-initiating cells. J. Clin. Investig..

[181] Howitt M.R., Lavoie S., Michaud M., Blum A.M., Tran S.V., Weinstock J.V., Gallini C.A., Redding K., Margolskee R.F., Osborne L.C. (2016). Tuft cells, taste-chemosensory cells, orchestrate parasite type 2 immunity in the gut. Science.

[182] Worthington J.J. (2015). The intestinal immunoendocrine axis: Novel cross-talk between enteroendocrine cells and the immune system during infection and inflammatory disease. Biochem. Soc. Trans..

[183] Bellono N.W., Bayrer J.R., Leitch D.B., Castro J., Zhang C., O’Donnell T.A., Brierley S.M., Ingraham H.A., Julius D. (2017). Enterochromaffin Cells Are Gut Chemosensors that Couple to Sensory Neural Pathways. Cell.

[184] Gross E.R., Gershon M.D., Margolis K.G., Gertsberg Z.V., Li Z., Cowles R.A. (2012). Neuronal serotonin regulates growth of the intestinal mucosa in mice. Gastroenterology.

[185] Zhao C.M., Hayakawa Y., Kodama Y., Muthupalani S., Westphalen C.B., Andersen G.T., Flatberg A., Johannessen H., Friedman R.A., Renz B.W. (2014). Denervation suppresses gastric tumorigenesis. Sci. Transl. Med..

[186] Greig C.J., Cowles R.A. (2017). Muscarinic acetylcholine receptors participate in small intestinal mucosal homeostasis. J. Pediatr. Surg..

[187] Neunlist M., Aubert P., Bonnaud S., Van Landeghem L., Coron E., Wedel T., Naveilhan P., Ruhl A., Lardeux B., Savidge T. (2007). Enteric glia inhibit intestinal epithelial cell proliferation partly through a TGF-beta1-dependent pathway. Am. J. Physiol. Gastrointest. Liver Physiol..

[188] Bach-Ngohou K., Mahe M.M., Aubert P., Abdo H., Boni S., Bourreille A., Denis M.G., Lardeux B., Neunlist M., Masson D. (2010). Enteric glia modulate epithelial cell proliferation and differentiation through 15-deoxy-12,14-prostaglandin J2. J. Physiol..

[189] Seguella L., Gulbransen B.D. (2021). Enteric glial biology, intercellular signalling and roles in gastrointestinal disease. Nat. Rev. Gastroenterol. Hepatol..

[190] Vicentini F.A., Keenan C.M., Wallace L.E., Woods C., Cavin J.B., Flockton A.R., Macklin W.B., Belkind-Gerson J., Hirota S.A., Sharkey K.A. (2021). Intestinal microbiota shapes gut physiology and regulates enteric neurons and glia. Microbiome.

[191] Sharkey K.A. (2015). Emerging roles for enteric glia in gastrointestinal disorders. J. Clin. Investig..

[192] Fukudo S., Kanazawa M., Kano M., Sagami Y., Endo Y., Utsumi A., Nomura T., Hongo M. (2002). Exaggerated motility of the descending colon with repetitive distention of the sigmoid colon in patients with irritable bowel syndrome. J. Gastroenterol..

[193] Kern F., Almy T.P., Abbot F.K., Bogdonoff M.D. (1951). The motility of the distal colon in nonspecific ulcerative colitis. Gastroenterology.

[194] Brierley S.M., Linden D.R. (2014). Neuroplasticity and dysfunction after gastrointestinal inflammation. Nat. Rev. Gastroenterol. Hepatol..

[195] Jarret A., Jackson R., Duizer C., Healy M.E., Zhao J., Rone J.M., Bielecki P., Sefik E., Roulis M., Rice T. (2020). Enteric Nervous System-Derived IL-18 Orchestrates Mucosal Barrier Immunity. Cell.

[196] Lei C., Sun R., Xu G., Tan Y., Feng W., McClain C.J., Deng Z. (2022). Enteric VIP-producing neurons maintain gut microbiota homeostasis through regulating epithelium fucosylation. Cell Host Microbe.

[197] Ahrends T., Aydin B., Matheis F., Classon C.H., Marchildon F., Furtado G.C., Lira S.A., Mucida D. (2021). Enteric pathogens induce tissue tolerance and prevent neuronal loss from subsequent infections. Cell.

[198] De Vadder F., Grasset E., Manneras Holm L., Karsenty G., Macpherson A.J., Olofsson L.E., Backhed F. (2018). Gut microbiota regulates maturation of the adult enteric nervous system via enteric serotonin networks. Proc. Natl. Acad. Sci. USA.

[199] Muller P.A., Schneeberger M., Matheis F., Wang P., Kerner Z., Ilanges A., Pellegrino K., Del Marmol J., Castro T.B.R., Furuichi M. (2020). Author Correction: Microbiota modulate sympathetic neurons via a gut-brain circuit. Nature.

[200] Collins J., Borojevic R., Verdu E.F., Huizinga J.D., Ratcliffe E.M. (2014). Intestinal microbiota influence the early postnatal development of the enteric nervous system. Neurogastroenterol. Motil..

[201] Anitha M., Vijay-Kumar M., Sitaraman S.V., Gewirtz A.T., Srinivasan S. (2012). Gut microbial products regulate murine gastrointestinal motility via Toll-like receptor 4 signaling. Gastroenterology.

[202] Hung L.Y., Boonma P., Unterweger P., Parathan P., Haag A., Luna R.A., Bornstein J.C., Savidge T.C., Foong J.P.P. (2019). Neonatal Antibiotics Disrupt Motility and Enteric Neural Circuits in Mouse Colon. Cell Mol. Gastroenterol. Hepatol..

[203] Brun P., Giron M.C., Qesari M., Porzionato A., Caputi V., Zoppellaro C., Banzato S., Grillo A.R., Spagnol L., De Caro R. (2013). Toll-like receptor 2 regulates intestinal inflammation by controlling integrity of the enteric nervous system. Gastroenterology.

[204] Burgueno J.F., Barba A., Eyre E., Romero C., Neunlist M., Fernandez E. (2016). TLR2 and TLR9 modulate enteric nervous system inflammatory responses to lipopolysaccharide. J. Neuroinflammation.

[205] Barki N., Bolognini D., Borjesson U., Jenkins L., Riddell J., Hughes D.I., Ulven T., Hudson B.D., Ulven E.R., Dekker N. (2022). Chemogenetics defines a short-chain fatty acid receptor gut-brain axis. Elife.

[206] Alemi F., Poole D.P., Chiu J., Schoonjans K., Cattaruzza F., Grider J.R., Bunnett N.W., Corvera C.U. (2013). The receptor TGR5 mediates the prokinetic actions of intestinal bile acids and is required for normal defecation in mice. Gastroenterology.

[207] McDole J.R., Wheeler L.W., McDonald K.G., Wang B., Konjufca V., Knoop K.A., Newberry R.D., Miller M.J. (2012). Goblet cells deliver luminal antigen to CD103+ dendritic cells in the small intestine. Nature.

[208] Chesne J., Cardoso V., Veiga-Fernandes H. (2019). Neuro-immune regulation of mucosal physiology. Mucosal Immunol..

[209] Knoop K.A., Gustafsson J.K., McDonald K.G., Kulkarni D.H., Coughlin P.E., McCrate S., Kim D., Hsieh C.S., Hogan S.P., Elson C.O. (2017). Microbial antigen encounter during a preweaning interval is critical for tolerance to gut bacteria. Sci. Immunol..

[210] Hirota C.L., McKay D.M. (2006). Cholinergic regulation of epithelial ion transport in the mammalian intestine. Br. J. Pharmacol..

[211] Takahashi T., Ohnishi H., Sugiura Y., Honda K., Suematsu M., Kawasaki T., Deguchi T., Fujii T., Orihashi K., Hippo Y. (2014). Non-neuronal acetylcholine as an endogenous regulator of proliferation and differentiation of Lgr5-positive stem cells in mice. FEBS J..

[212] Takahashi T., Shiraishi A., Murata J. (2018). The Coordinated Activities of nAChR and Wnt Signaling Regulate Intestinal Stem Cell Function in Mice. Int. J. Mol. Sci..

[213] Talbot J., Hahn P., Kroehling L., Nguyen H., Li D., Littman D.R. (2020). Feeding-dependent VIP neuron-ILC3 circuit regulates the intestinal barrier. Nature.

[214] Yan Y., Ramanan D., Rozenberg M., McGovern K., Rastelli D., Vijaykumar B., Yaghi O., Voisin T., Mosaheb M., Chiu I. (2021). Interleukin-6 produced by enteric neurons regulates the number and phenotype of microbe-responsive regulatory T cells in the gut. Immunity.

[215] Jodal M., Wingren U., Jansson M., Heidemann M., Lundgren O. (1993). Nerve involvement in fluid transport in the inflamed rat jejunum. Gut.

[216] Ghoshal U.C., Gwee K.A. (2017). Post-infectious IBS, tropical sprue and small intestinal bacterial overgrowth: The missing link. Nat. Rev. Gastroenterol. Hepatol..

[217] Obermayr F., Hotta R., Enomoto H., Young H.M. (2013). Development and developmental disorders of the enteric nervous system. Nat. Rev. Gastroenterol. Hepatol..

[218] Holland A.M., Bon-Frauches A.C., Keszthelyi D., Melotte V., Boesmans W. (2021). The enteric nervous system in gastrointestinal disease etiology. Cell Mol. Life Sci..

[219] Feldman E.L., Callaghan B.C., Pop-Busui R., Zochodne D.W., Wright D.E., Bennett D.L., Bril V., Russell J.W., Viswanathan V. (2019). Diabetic neuropathy. Nat. Rev. Dis. Primers.

[220] Travagli R.A., Browning K.N., Camilleri M. (2020). Parkinson disease and the gut: New insights into pathogenesis and clinical relevance. Nat. Rev. Gastroenterol. Hepatol..

[221] Geng Z.H., Zhu Y., Li Q.L., Zhao C., Zhou P.H. (2022). Enteric Nervous System: The Bridge Between the Gut Microbiota and Neurological Disorders. Front. Aging Neurosci..

[222] Eicher A.K., Kechele D.O., Sundaram N., Berns H.M., Poling H.M., Haines L.E., Sanchez J.G., Kishimoto K., Krishnamurthy M., Han L. (2022). Functional human gastrointestinal organoids can be engineered from three primary germ layers derived separately from pluripotent stem cells. Cell Stem Cell.

